# Oligodendrocyte progenitor cells' fate after neonatal asphyxia—Puzzling implications for the development of hypoxic–ischemic encephalopathy

**DOI:** 10.1111/bpa.13255

**Published:** 2024-03-19

**Authors:** Justyna Janowska, Justyna Gargas, Karolina Zajdel, Michal Wieteska, Kamil Lipinski, Malgorzata Ziemka‐Nalecz, Malgorzata Frontczak‐Baniewicz, Joanna Sypecka

**Affiliations:** ^1^ Department of NeuroRepair Mossakowski Medical Research Institute PAS Warsaw Poland; ^2^ NOMATEN Center of Excellence, National Center for Nuclear Research Otwock Poland; ^3^ Electron Microscopy Research Unit Mossakowski Medical Research Institute PAS Warsaw Poland; ^4^ Small Animal Magnetic Resonance Imaging Laboratory Mossakowski Medical Research Institute PAS Warsaw Poland; ^5^ Division of Nuclear and Medical Electronics Warsaw University of Technology Warsaw Poland

**Keywords:** MRI, neonatal asphyxia, oligodendrocytes, OPCs, TEM, white matter injury

## Abstract

Premature birth or complications during labor can cause temporary disruption of cerebral blood flow, often followed by long‐term disturbances in brain development called hypoxic–ischemic (HI) encephalopathy. Diffuse damage to the white matter is the most frequently detected pathology in this condition. We hypothesized that oligodendrocyte progenitor cell (OPC) differentiation disturbed by mild neonatal asphyxia may affect the viability, maturation, and physiological functioning of oligodendrocytes. To address this issue, we studied the effect of temporal HI in the in vivo model in P7 rats with magnetic resonance imaging (MRI), microscopy techniques and biochemical analyses. Moreover, we recreated the injury in vitro performing the procedure of oxygen–glucose deprivation on rat neonatal OPCs to determine its effect on cell viability, proliferation, and differentiation. In the in vivo model, MRI evaluation revealed changes in the volume of different brain regions, as well as changes in the directional diffusivity of water in brain tissue that may suggest pathological changes to myelinated neuronal fibers. Hypomyelination was observed in the cortex, striatum, and CA3 region of the hippocampus. Severe changes to myelin ultrastructure were observed, including delamination of myelin sheets. Interestingly, shortly after the injury, an increase in oligodendrocyte proliferation was observed, followed by an overproduction of myelin proteins 4 weeks after HI. Results verified with the in vitro model indicate, that in the first days after damage, OPCs do not show reduced viability, intensively proliferate, and overexpress myelin proteins and oligodendrocyte‐specific transcription factors. In conclusion, despite the increase in oligodendrocyte proliferation and myelin protein expression after HI, the production of functional myelin sheaths in brain tissue is impaired. Presented study provides a detailed description of oligodendrocyte pathophysiology developed in an effect of HI injury, resulting in an altered CNS myelination. The described models may serve as useful tools for searching and testing effective of effective myelination‐supporting therapies for HI injuries.

## Graphical abstract

This study presents the effect of neonatal hypoxia–ischemia on white matter development. Study was conducted with different imaging techniques on rat model of the injury, providing evidences that oligodendrocyte differentiation or maturation in hypoxic–ischemic brain is affected. Results were supported by studies on an in vitro model using primary rat oligodendrocyte progenitor cells.

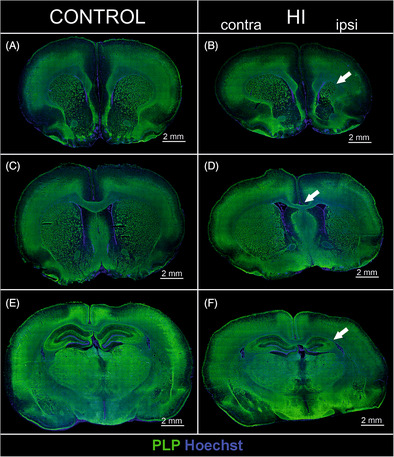

## 1 INTRODUCTION

According to the World Health Organization, neonatal asphyxia is one of the main reasons for the death of new‐born babies [1]. It is often caused by a preterm or complicated delivery, a mother's infection, or hypotension [2, 3]. The consequence of asphyxia is hypoxic–ischemic (HI) injury that can affect many organs, such as the heart, liver, and kidneys, which is evidenced in clinical cases [4, 5], as well as in animal models [6]. In case of these, the pathophysiological changes are often reversible and do not lead to long‐term consequences. In contrast, the central nervous system is particularly affected. Approximately 1–8 cases per 1000 live births develop hypoxic–ischemic encephalopathy (HIE) [7]. This condition is diagnosed primarily by magnetic resonance imaging (MRI), which detects abnormalities of the CNS [8], such as diffuse damage within gray and white matter (mostly in children born prematurely [9]) and changes in the basal ganglia and thalamus (mostly in full‐term children born with HI complications [10]). Neurodevelopmental processes of children with mild asphyxia are usually normal, yet cognitive or motor deficits may appear [11, 12]. However, moderate to severe asphyxia contributes to the development of cerebral palsy and epilepsy and may also increase the risk of developing autism spectrum disorders [13, 14, 15].

The white matter of CNS is formed by clusters of axons and dendrites of neurons that are surrounded by myelin sheath, produced by cells called oligodendrocytes. Myelin sheath is an excellent electrical insulator that enables fast propagation of nerve impulses and creates a necessary protective barrier for axons [16]. The process of myelination occurring under physiological conditions is well described in the literature thanks to, in part, studies using animal models. This has been particularly contributed to by the studies of Craig et al., who characterized the similarities in white matter development in the rat and humans. With this knowledge, researchers are better able to understand what factors may interfere with oligodendrocyte growth in different models of perinatal brain injury [17]. During the development of the CNS, oligodendrocytes differentiate from oligodendrocyte progenitor cells (OPCs). OPCs have been shown to generate in distinct weaves, starting as early as around 12.5 days of embryonic life in rat (E12.5). A second wave migrates from the dorsal ventricular zone approximately at E.14–E.15, while the next weave is generated postnatally. The recent bulk and single‐cell analyses indicate, however, that OPCs which originated from distinct spatial and temporal brain regions acquire the similar transcriptional identity around postnatal Day 7 (P7) [18, 19]. In humans, this process is initiated at 15 weeks of gestation (15 GW) respectively. OPCs migrate toward the cortex in response to a gradient of concentrations of chemotactic factors present in the tissue, such as bone morphogenic proteins (BMPs) and fibroblast growth factor (FGF). OPCs that populate their niche begin proliferation under the influence of PDGF (platelet‐derived growth factor), and once optimal cell density is reached in the tissue (around 20 GW), further differentiation begins. On P2 in rats and between 18 and 28 GW in humans, the developing white matter is dominated by late progenitors, also known as preoligodendrocytes (pre‐Ols) and immature oligodendrocytes (iOLs). Myelination begins at P7 in rats and between 30 and 36 GW in humans, respectively, when the first oligodendrocytes, producing myelin membrane‐building proteins such as proteolipid protein (PLP), myelin‐basic protein (MBP) or myelin‐associated glycoprotein (MAG) appear. At this time, however, iOLs are the most abundant group of oligodendrocytic cells. The degree of myelination of the rat CNS, similar to that of term‐born infants, is reached in the second week of life (P14) in rats [17, 20, 21, 22].

Maturation of progenitor cells is regulated by many extracellular stimuli, for example, growth factors, chemokines, interleukins, or ATP [23, 24, 25]; thus, the disruption of neural tissue homeostasis induced by neonatal asphyxia may also affect the growth of these cells and their later functioning. It seems to be especially important in case of complications occurring in the 30–36th week of pregnancy when OPCs and immature oligodendrocytes are the most abundant group of oligodendrocytes inhabiting the developing CNS. The studies indicate that among the oligodendrocyte lineage cells, the fraction of cells in a late stage of differentiation is the most sensitive to factors that may induce necrosis or apoptosis [26]. So far, the potential contribution of phenomena such as glial scar formation [27, 28] and increased expression of selected trophic factors has been shown to inhibit the maturation of OPCs [24, 29, 30].

Currently, there are no sufficiently effective therapeutic strategies to restore the nervous tissue and thus, to promote the normal development of the CNS. The only commonly used practice is the application of therapeutic hypothermia during the first few hours of life, which suppresses brain damage by inhibiting the process of neural cell apoptosis and decreasing inflammatory response [31]. However, positive effects of this procedure are observed only in moderate and mild HIE [32]. What is additionally worth noting, is that long‐term observations of patients who have received therapeutic hypothermia still indicate an impaired white matter development and the occurrence of neurodevelopmental problems in school‐age children [33, 34].

In the following study, we were interested in finding the effect of mild neonatal HI in the rat in vivo model of the injury, on the fate of oligodendrocytes at early stages of differentiation, whether they are sensitive to the injury and capable of maturation and production of myelin proteins. We hypothesized that OPC differentiation disturbed by mild neonatal asphyxia may affect the viability, maturation, and physiological functioning of oligodendrocytes, resulting in deficient production of myelin sheaths building the white matter. In the in vitro model of the injury, with primary rat OPC cultures subjected to oxygen–glucose deprivation (OGD), we wanted to verify whether the temporary restriction of oxygen and nutrients can itself affect cell maturation. Identifying all the pathophysiological mechanisms for the development of white matter damage after neonatal HI will help define new therapeutic targets.

## 2 MATERIALS AND METHODS

All experiments were performed using Wistar rats of both sexes. The procedures the animals underwent in the experiments were approved by the fourth Local Ethical Committee (approval numbers 39/2015 and 83/2015). The number of animals used in the study was 31 for the in vivo studies and 72 for the in vitro studies.

### 2.1 The in vivo model of neonatal HI

**FIGURE 1:**
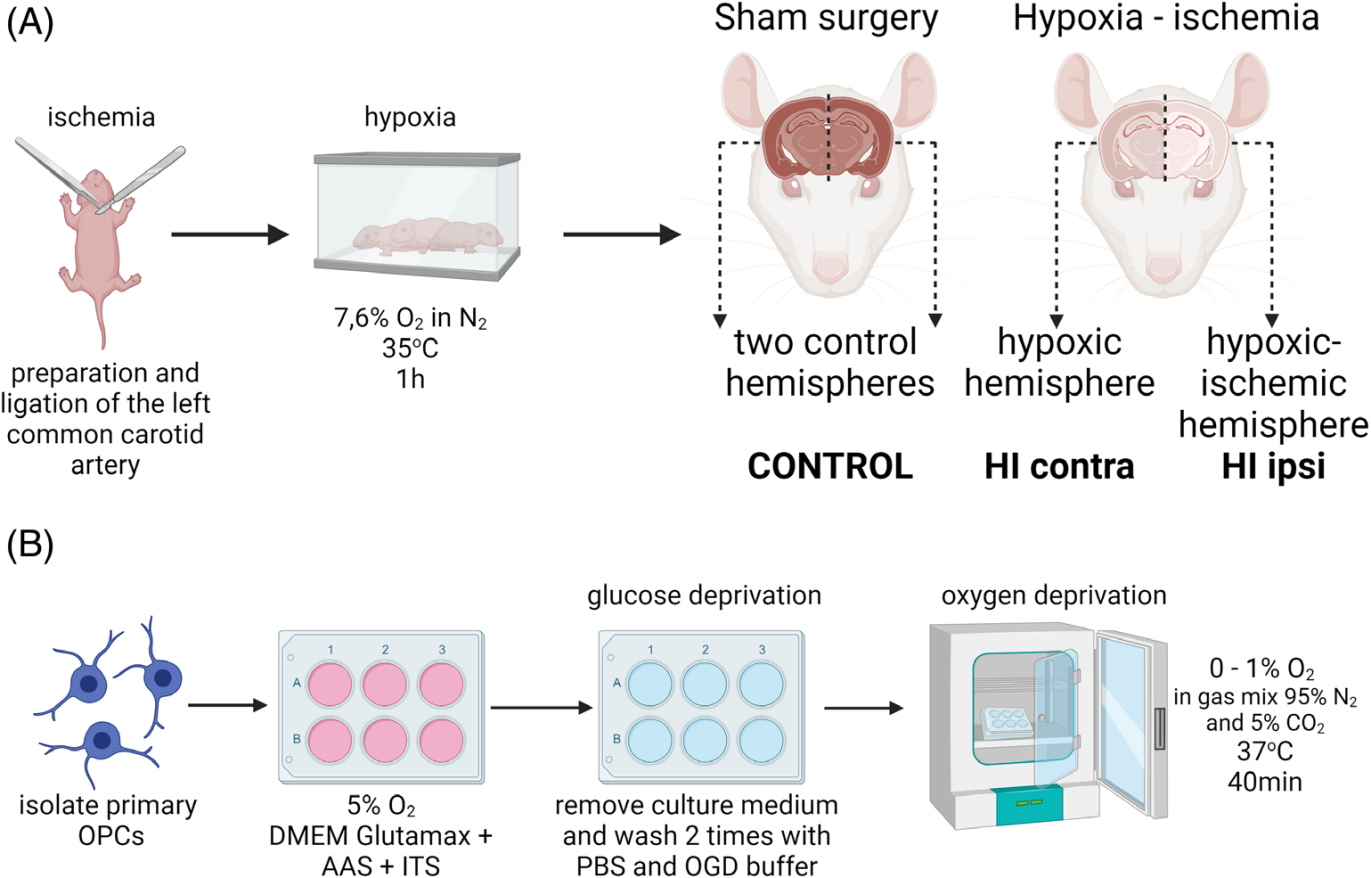
Schematic representations of experimental models used in the study. (A) Hypoxic–ischemic injury in vivo. (B) Oxygen–glucose deprivation to OPCs in vitro. OPC, oligodendrocyte progenitor cell. Figure created with BioRender.com.

HI was induced in 7‐day‐old rats, according to a previously published procedure [35, 36]. Rats from each litter size 10–12, with an average sex distribution corresponding to 45.6% of male pups and 54.4% of female pups, were randomly selected into groups (control or HI). Pups were anesthetized with inhaled isoflurane in oxygen (induction 4% and maintenance 2%). In the HI group, the left common carotid artery was prepared and ligated, inducing unilateral cerebral ischemia. Rats were caged with the dam for 1–1.5 h and then placed in a hypoxic chamber (7.6% O_2_ in N_2_, 33°C) for 1 h. Control animals underwent sham surgery only, when animals were anesthetized and an artery was exposed only. After the treatments, control and HI animals from the same litter were returned to the cage with their dam. Animals were kept in the breeding room of the animal house, at a temperature of 22 ± 1°C, 12/12 h light/dark cycle. All animals survived to the planned endpoint. Before collecting brain hemispheres for analyses, rats were anesthetized with isoflurane (4%), followed by decapitation. First time point to sacrifice animals was scheduled for 3 days after HI (P10). For later time points (4, 9, and 10 weeks after HI), animals were separated from their mothers at P21. Then they were placed two individuals each in a cage, with free access to water and food ad libitum. All experiments were performed and samples were collected within 15 months of the study. The obtained material was divided into three groups: control (or CT; sham surgery), HI contra (contralateral hemisphere to the induced injury, which became hypoxic only), and HI ipsi (ipsilateral hemisphere to the induced injury; HI), as shown in Figure 1A. Samples of brain hemispheres without visible damage to HI ipsi hemisphere were excluded.

### 2.2 The in vitro model—Primary cultures of OPCs subjected to OGD

#### 2.2.1 Isolation and culture of OPCs

OPCs were derived from primary rat mixed glial cultures according to a method described elsewhere [37, 38]. Cerebral hemispheres of 2‐day‐old rats were mechanically dissociated in culture medium consisting of high glucose DMEM Glutamax (Gibco), 10% fetal bovine serum (FBS; Gibco), and 1% AAS (antibiotic–antimycotic solution; Sigma‐Aldrich) and plated onto culture flasks (Nunclon EasY T75) coated with poly‐l‐lysine (Sigma). For the following 11–13 days, obtained mixed glial cultures were maintained under standard in vitro culture conditions (37°C, 21% O_2_, 5% CO_2_) with medium change every 2–3 days. OPCs were then isolated using a mechanical method of shaking, that takes use of the different adhesive properties of the various glial fractions. Flasks with mixed glial cultures were stacked on an orbital shaker operating at 160 rpm and placed in an incubator. After 1 h of shaking, the culture medium was changed for fresh and the shaking continued for the next 18–20 h, when the OPCs gently detached from the culture medium. Obtained supernatant was centrifuged for 5 min at 1200 rpm, washed with phosphate‐buffered saline (PBS, Gibco), and centrifuged again. The cells obtained were resuspended in serum‐free culture medium (DMEM Glutamax high glucose [Gibco], 1% ITS [insulin‐transferrin‐selenium supplement; Gibco], and 1% AAS [Sigma‐Aldrich]) and seeded at a density of approximately 3 × 10^4^ cells/cm^2^ into 24‐well plates (Nunclon, ThermoFisher) with poly‐l‐lysine‐coated slides for immunofluorescence staining or poly‐l‐lysine‐coated six‐well plates (Nunclon, ThermoFisher) for biochemical assays. The OPCs were cultured under physiological normoxia (37°C, 5% O_2_, 5% CO_2_).

#### 2.2.2 OGD procedure

To induce HI damage under culture conditions, a modified OGD procedure was used [37, 39]. The method involves temporarily replacing the culture medium with a buffer solution of the composition: 124 mM NaCl, 4.8 mM KCl, 1.2 mM KH_2_PO_4_, 1.5 mM CaCl_2_, 1 mM MgCl_2_, 10 mM mannitol, and 25 mM NaHCO_3_. Buffer was treated with a stream of N_2_ to remove dissolved oxygen molecules before use. OPC cultures, about 4 h after seeding, when cells had time to adhere to the culture dish, were washed with PBS and then with OGD buffer, followed by replacement of the medium in the cell cultures with the buffer. To achieve hypoxia, a multi‐gas incubator was used in which the oxygen concentration during the OGD procedure was 0%–1%, CO_2_ concentration was 5%, and the remaining was N_2_. From reaching an oxygen level of 0.9% (approximately 10 min), a hypoxia time of 40 min was measured (Figure 1B). After this time in control and OGD‐treated cultures, the culture medium was replaced with the same volume as the fresh one.

### 2.3 Magnetic resonance imaging

MRI was performed for each animal (control *N* = 2; post‐HI *N* = 3) at two time points: 2 and 10 weeks after HI. The rats underwent isoflurane anesthesia throughout the procedure (induction 4%, maintenance 1.5%–2%). The animals were positioned inside a 7 T MRI scanner (model BioSpec 70/30 USR, Bruker Biospin, Ettlingen, Germany) on a scanning bed in a supine position. The body temperature and respiratory rate were supervised using a small animal monitoring system (model 1025, SA Instruments). A phased array (2 × 2 elements) receive coil (model 1P T11483V3, Bruker Biospin, Ettlingen, Germany) was placed on top of the animal's head for signal reception. A volumetric transmit coil (diameter of 86 mm, model 1P T12053V3, Bruker Biospin, Ettlingen, Germany) was used for radio‐frequency (RF) excitation.

A single scanning session consisted of a localizer scan, a shimming procedure, anatomical imaging, and diffusion tensor imaging (DTI). Anatomical image of the whole brain was acquired using a T2‐weighted RARE sequence with the following parameters: effective echo time (TE) = 30 ms, repetition time (TR) = 5000 ms, RARE factor = 4, spatial resolution = 0.125 × 0.125 × 0.5 mm^3^, number of slices = 54, and number of averages = 3. DTI scans were performed using an echo planar imaging (EPI) sequence with five baseline images and 30 gradient directions (*b* value = 670 s/mm^2^, TE = 33 ms, TR = 4000 ms, spatial resolution = 0.156 × 0.234 × 1.0 mm^3^, number of slices = 16, and number of averages = 1).

All data processing (including the generation of DTI parametric maps) and pre‐processing were performed using MRTrix 3 Software [40]. Pre‐processing included MP‐PCA denoising and Gibbs ringing removal. Motion and distortion correction were performed using FSL algorithms [41]. Bias field correction was performed using ANTS [42]. Automatic structure segmentation was performed by realigning the SIGMA Rat Brain Template [43] to match each of the individual images. T2‐weighted images were used for whole‐brain structure volumetry, whereas the DTI scans were used for calculating fractional anisotropy (FA) statistics for structures of interest.

### 2.4 Fixing tissue and cells for imaging with fluorescence microscopy techniques

At P10 (3 days after HI; control *N* = 3; post‐HI *N* = 5) and P77 (10 weeks after HI; control *N* = 3; post‐HI *N* = 5), brains were isolated and frozen immediately at −80°C. Brains were serially sliced in the frontal plane into 10‐μm thick slices on a Microm HM550 cryostat (Thermo Scientific). The sections were placed on silane‐coated glass slides. Selected slices, covering the hippocampal region and striatum, were dried for 1–2 h at room temperature and then fixed for 15 min in a solution of 4% paraformaldehyde in PBS. Slides were washed three times in PBS, followed by an immunofluorescence staining procedure. For each staining, one to three slides obtained from each experimental animal were analyzed from frontal sections of the brain including the striatum and hippocampal formation.

Cell cultures were fixed on Days 1 and 3 after OGD. Culture medium was removed from the cultures, washed two times with PBS buffer, fixed in a solution of 4% paraformaldehyde in PBS at room temperature for 15 min, and washed two times with PBS.

### 2.5 The evaluation of oligodendrocyte maturation and proliferation using immunofluorescence staining

Fixed tissues and cells were immunolabeled according to the procedure described in detail in the Supporting Information. Briefly, preparations were blocked and incubated with the primary antibody overnight, then washed and labeled with fluorescent secondary antibody. To visualize cell nuclei, Hoechst 33258 (Sigma‐Aldrich; 1:150 in PBS) was applied. The slides were sealed with a coverslip or glass slide using a Fluorescence Mounting Medium reagent (Dako). Microscopic images were acquired with LSM 780 microscope (Zeiss). A detailed description of collecting images for analysis and the course of analysis can be found in the Supporting Information.

### 2.6 The evaluation of cell proliferation in the in vitro culture using Ki67 staining and BrdU incorporation assay

The first method to estimate the number of dividing cells was the double immunofluorescence labeling using an antibody that detects the proliferation marker, Ki67 protein, present on the surface of chromosomes during mitotic division, along with oligodendrocyte marker OLIG2 (Supporting Information, Table 1). The second method was the BrdU (bromodeoxyuridine) incorporation assay which allows the labeling of all cells that have resulted from cell divisions since the addition of this reagent to the culture medium. After OGD on cells cultured in 24‐well plates with slides, the buffer and medium in the control cultures were exchanged with medium supplemented with 5 μM BrdU (Sigma‐Aldrich) and maintained for 3 days. The BrdU was incorporated into the DNA during the replication process and was then detected using a specific antibody. The cultures were washed three times with PBS buffer, then fixed in 4% paraformaldehyde for 15 min and incubated in 2 N HCl at 37°C for 1 h. After this time, the cultures were washed three times for 10 min each with borate buffer (0.2 M boric acid; 0.05 M borax, pH 8.4) and then three times with PBS solution. Slides were then labeled with anti‐PDGFRα antibody, as described above. Microscopic images were acquired with LSM 780 (Zeiss) microscope. Cells were counted manually with ImageJ software.

### 2.7 Transmission electron microscopy

The efficiency of myelination after HI insult was assessed by examination of myelin ultrastructure using transmission electron microscopy (TEM). Rats at P70 (9 weeks after HI; *N* = 5) were anesthetized and gently perfused with fixative solution composed of 2% paraformaldehyde (Serva), 2.5% glutaraldehyde (Agar Scientific), and 0.1 M cacodylate buffer (Agar Scientific), pH 7.4 at 4°C. The selected brain regions (striatum and corpus callosum) were isolated and additionally fixed overnight in the same fixative solution. Collected samples were washed three times in 0.1 M cacodylate buffer and post‐fixed in 1% osmium tetraoxide (Serva) and 0.8% potassium ferricyanide (Alchem) for 2 h. The post‐fixed brain samples were dehydrated in an ascending alcohol series from 30% up to 100% and propylene oxide. Next, the tissues were embedded in epoxy resin blocks and polymerized at 60°C for 24 h. The ultrathin sections (~60 nm) were cut on an MTXL ultramicrotome (RMC, USA) using a diamond knife (Ultra 45°, Diatome, USA), placed on copper grids (300 mesh, Electron Microscopy Sciences), and post‐stained used the double contrast method with uranyl acetate (Serva; 30 min) and lead citrate (Agar Scientific; 15 min). The myelin ultrastructure analysis was performed using a JEM‐1011 TEM (JEOL, Japan) operated at 80 kV accelerating voltage. To assess the myelin sheath thickness in selected brain regions, electronograms under a magnification of 12,000× were taken. G‐ratio was evaluated by means of ratio of inner myelin diameter to outer myelin diameter with MyelTracer software [44]. Only the cross‐sections perfectly perpendicular to the long axis of myelin were evaluated.

### 2.8 ELISA‐based quantification of myelin proteins

For ELISA‐based quantification of myelin proteins, brain hemispheres were isolated 4 weeks after HI rats (P35; control *N* = 4; post‐HI *N* = 6), frozen immediately after isolation, and stored at −80°C. Prior to homogenate preparation, the hippocampus was extracted from the brain hemispheres. The brain hemispheres without hippocampus and hippocampi were analyzed separately. The tissues were homogenized in a handheld glass homogenizer at 4°C using CelLyticM buffer (1 mL/100 mg tissue; Sigma‐Aldrich) with Protease Inhibitor Cocktail (1:100; Sigma‐Aldrich). Total protein concentration in tissue homogenates was measured using a modified Lowry method with the DC Protein Assay kit (Bio‐Rad). For the standard curve, bovine serum albumin standard solutions (Sigma‐Aldrich) were used. The concentrations of selected proteins in the samples were determined using commercially available ELISA kits according to protocols provided by the manufacturers, including Rat Myelin Basic Protein [MBP] ELISA Kit, abbexa, abx052948; Rat Proteolipid Protein 1 [PLP1] ELISA Kit, abbexa, abx512900; Rat MAG ELISA Kit, RayBiotech, P07722. Absorbance readings were performed on a FluoStar Omega spectrophotometer (BMG Labtech). To normalize the results obtained by ELISA, the concentrations obtained were related to the concentration of total protein in the sample.

### 2.9 Cell viability assays

#### 2.9.1 alamarBlue

The viability of cells in culture after OGD was estimated based on their ability to convert the weakly fluorescent substrate of the alamarBlue reagent (resazurin) into a strongly fluorescent product (resorufin). This process depends on both the number of cells and their metabolic activity. At 1, 3, and 6 days after OGD, the medium was replaced with fresh medium supplemented with 10% alamarBlue reagent (Invitrogen). After 3 h of incubation, fluorescence was measured using a Fluoroskan Ascent FL reader (Labsystems), and then the medium was replaced with fresh medium or the cultures were fixed. Results were normalized with the number of plated cells per well and with the value of fluorescence measured in the first analyzed time point.

#### 2.9.2 Lactate dehydrogenase assay

The amount of lactate dehydrogenase (LDH) released into the culture medium along with cell damage and changes in cell membrane integrity were measured using the LDH‐Glo Assay kit (Promega), which uses a specific bioluminescence reaction. Samples of 10 μL were taken from the culture medium, diluted 25‐fold in an appropriate buffer that retains the enzyme activity, and frozen at 80°C until ass samples were collected. Media samples were collected immediately after OGD (in this case, OGD buffer was collected), and 1, 4, 24, and 48 h after OGD. The reaction was performed in a 96‐well plate according to the protocol provided by the manufacturer. Bioluminescence was read using a Fluostar Omega spectrophotometer (BMG Labtech). Results were normalized with the value of luminescence measured in the first analyzed time point.

### 2.10 RT‐qPCR gene expression analysis

To evaluate the expression of selected oligodendrocyte differentiation genes under OGD, RT‐qPCR (reverse transcription‐quantitative polymerase chain reaction) was used. The isolation of total RNA from cells on Days 1 and 3 after OGD was performed with the NucleoSpin RNA Plus XS kit (Macherey‐Nagel) according to the manufacturer's protocol. The concentration and purity of RNA in the obtained samples were determined using a NanoDrop One spectrophotometer (ThermoFisher). In the next step, a reverse transcription reaction was performed using 500 ng of RNA sample using iScript cDNA Synthesis Kit (Bio‐Rad). The reaction was performed using an Eppendorf Mastercycler pro vapo.protect thermocycler according to the following protocol: priming (5 min/25°C), reverse transcription (20 min/46°C), and reverse transcriptase inactivation (1 min/95°C). Primers for the following analysis of gene expression changes using the qPCR technique, were designed with Primer‐BLAST using reference sequences from the NCBI (National Center for Biotechnology Information) database. The primer sequences for individual genes are listed in Supporting Information: Table 2. The primers were synthesized in the DNA Sequencing Laboratory of the Institute of Biochemistry and Biophysics of the Polish Academy of Sciences. The semi‐quantitative PCR (qPCR) reaction was performed using an optimized iTaq Universal SYBR Green Supermix reaction mixture (Bio‐Rad) and Applied Biosystems 7500 Fast thermocycler according to the following protocol: polymerase activation and DNA denaturation (20 s/95°C), 40 amplification cycles (denaturation 1 s/95°C; annealing and plate read 20 s/60°C), and melting curve analysis (65–95°C). Differences in gene expression were normalized with the B2M (beta‐2 microglobulin) and Rpl13 (ribosomal protein L13; ribosomal protein L13) genes using the qBase model, taking into account reaction efficiency and two reference genes [45].

### 2.11 Statistical analysis

In MRI results presented as heat maps, columns correspond to different time points and/or experimental groups and the rows in the graph correspond to different brain regions analyzed. Each square in the graph is color‐coded to denote the value according to the included scale. In results presented as scatter graphs, single dot represent an average result value obtained from one animal in the group, horizontal middle line represent the mean value, and whiskers represent the standard deviation. Bar plots represent mean values with error bars reflecting standard deviation. In box and whisker plots, the bottom edge of the box corresponds to the first quartile of the results obtained, the middle of the box corresponds to the median, the top edge of the box corresponds to the third quartile, whiskers represent the 10–90 percentile range of the values obtained, and values below and above the whiskers are drawn as individual points.

Statistical analyses of the results obtained from three to five biological replicates (for MRI and TEM 2–3) of a given experiment were performed using GraphPad PRISM 10.0 software. The normal distribution was tested with the Shapiro–Wilk test. Depending on the analyzed data, results were evaluated with two‐tailed paired on unpaired *T* test, one‐way or two‐way ANOVA with Tukey's correction, Pearson's correlation analysis, or non‐parametric tests (Kruskal–Wallis with Dunn's correction or Mann–Whitney test). Differences at *p* < 0.05 were considered statistically significant. Details of the test used can be found in the figure legends.

## 3 RESULTS

### 3.1 Evaluation of the effect of mild neonatal HI on development of white matter in the in vivo model

#### 3.1.1 Brain development after neonatal HI

**FIGURE 2:**
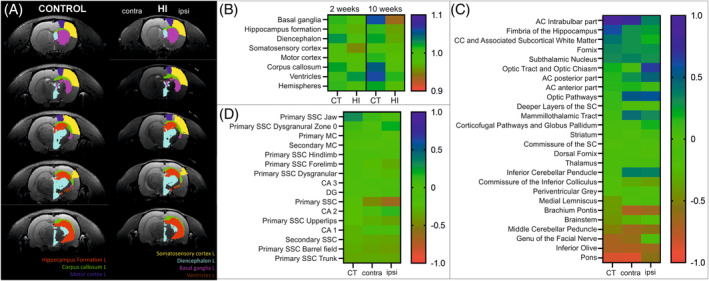
Results of MRI analysis performed 2 and 10 weeks after HI. (A) Representative scans collected 10 weeks after HI with color labels covering selected brain regions analyzed with volumetry. (B) Results of volumetry analysis, presented as the ratio between the volume of the left (L) to the right (R) brain region in control animals (*N* = 2), or HI ipsi to HI contra brain region in HI animals (*N* = 3), in two analyzed time points (2 and 10 weeks after HI) were evaluated with Shapiro–Wilk test for normal distribution and paired two‐tailed *T* tests. (C) Results of the analysis of fractional anisotropy (FA) measurements in white matter regions, presented as average difference in FA over time with the relation to 2 weeks values. (D) Results of the analysis of FA measurements in selected grey matter regions, presented as average difference in FA over time with the relation to 2 weeks values. AC, anterior commissure; CA, cornu ammonis; CC, corpus callosum; DG, dentate gyrus; MC, motor cortex; MRI, magnetic resonance imaging; SC, superior colliculus; SSC, somatosensory cortex.

The effect of neonatal HI injury on the development of CNS in rats was evaluated non‐invasively with MRI, performed 2 and 10 weeks after inducing the injury. Collected scans fitted to the Wistar rat brain atlas allowed the automatic segmentation and analysis of selected brain regions by means of changes in volume and diffusivity, which is often used to evaluate white matter development and injury. Figure 2A shows sample brain sections of CT and HI animals with color labels overlaid on the left hemisphere. These regions were subjected to the volumetric analysis, with results presented in Figure 2B. Results of volumetry analysis are presented as the ratio between the volume of the left (L) to the right (R) brain region in control animals, or HI ipsi to HI contra brain region in HI animals, in two analyzed time points. Obtained data were analyzed with paired *t* test. Two weeks after injury there are already observed significant changes in volumes of analyzed structures (*p* = 0.0053), especially a decrease in volume of somatosensory cortex and hippocampus (average values below 1, red) and an increase in the volume of ventricles (average values above 1, blue) in HI ipsi hemisphere compared to HI contra. While there are differences in the volume of diencephalon and corpus callosum between the left and right hemispheres in control animals, these were the same volume in HI animals (values close to 1, green). During the following 8 weeks, in the same animals, the variations between the volume of basal ganglia, corpus callosum, and ventricles of control animals have become even more apparent. Contrary, in the ipsi hemispheres of HI animals, there was a significant hypotrophy of basal ganglia, hippocampi, and diencephalon (*p* = 0.001). Comparing the volumes between CT and HI animals, we observed a 4% reduction in the volume of whole brain in HI compared to control animals in two analyzed time points. And comparing hemispheres, there was a tissue loss in ipsi hemisphere versus control hemisphere by 5.2% and 6.1%, at 2 and 10 weeks after HI time points, respectively.

In the following study, we did not further directly measure tissue loss or neuronal damage, since the main interest of this research has been focused on white matter development. However, in the previous studies conducted on the same animal model in our group, we examined brain damage 7 and 14 days after HI [36, 46, 47, 48]. We showed the loss of neurons and signs of cerebral edema with swollen cells throughout the frontal cortex and the hippocampus in the ipsilateral hemisphere as well as an activation of immunological response 6–7 days after HI [46, 48]. Observations performed 14 days after the injury revealed an atrophy of the ipsilateral hemisphere and brain asymmetry as well as an enlargement of the lateral ventricles [36, 47]. However, it should be noted that this model of HI can produce different degrees of brain damage reflecting the individual response of the animals to the insult. Changes in white matter ultrastructure as well as grey matter were assessed based on the changes in FA between 10 and 2 weeks after the injury within the same treated hemisphere. While in the white matter of CT hemispheres, there is an increase in FA (values close to 1, blue) within fiber tracts of anterior commissure, fimbria of the hippocampus as well as corpus callosum, there are minor changes within these structures in HI contra and HI ipsi hemispheres of animals after the injury (values close to 0, green), which may indicate disturbed white matter development (Figure 2C). Interestingly, there is an increase in FA within other white matter regions, for example optic tract and optic pathways. Analyses of changes in FA during growth also revealed a decrease in FA in selected regions (values close to −1, red), both in control and HI animals. Grey matter structures did not change in terms of FA during the growth of animals between 2 and 10 weeks after HI (Figure 2D).

#### 3.1.2 Myelination after neonatal HI evaluated with immunofluorescence microscopy

**FIGURE 3:**
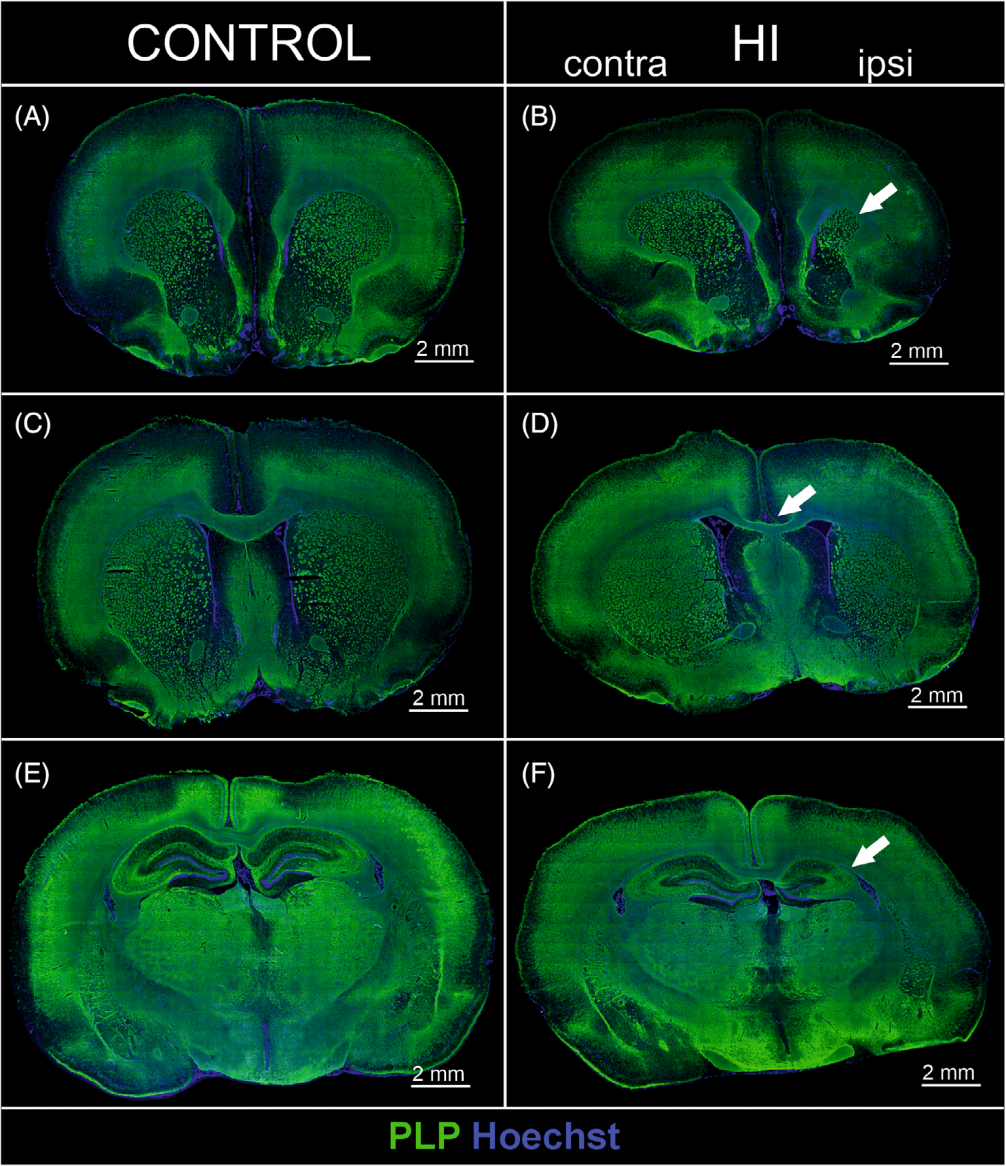
Overview image of brain development 10 weeks after HI. Slices were stained with anti‐PLP (green) to visualize myelin and Hoechst (blue) to label cell nuclei. Control (A) and HI (B) slices covering striatum, with an arrow presenting the reduced volume of this structure in the HI ipsi hemisphere in comparison to HI contra as well as control hemispheres. Control (C) and HI (D) slices covering corpus callosum with an arrow indicating thinner corpus callosum in HI tissue compared to control. Control (E) and HI (F) slices covering hippocampus; white arrow indicates significant hypotrophy of this structure within HI ipsi hemisphere. HI, hypoxia–ischemia. Scale bar corresponds to 2 mm.

**FIGURE 4:**
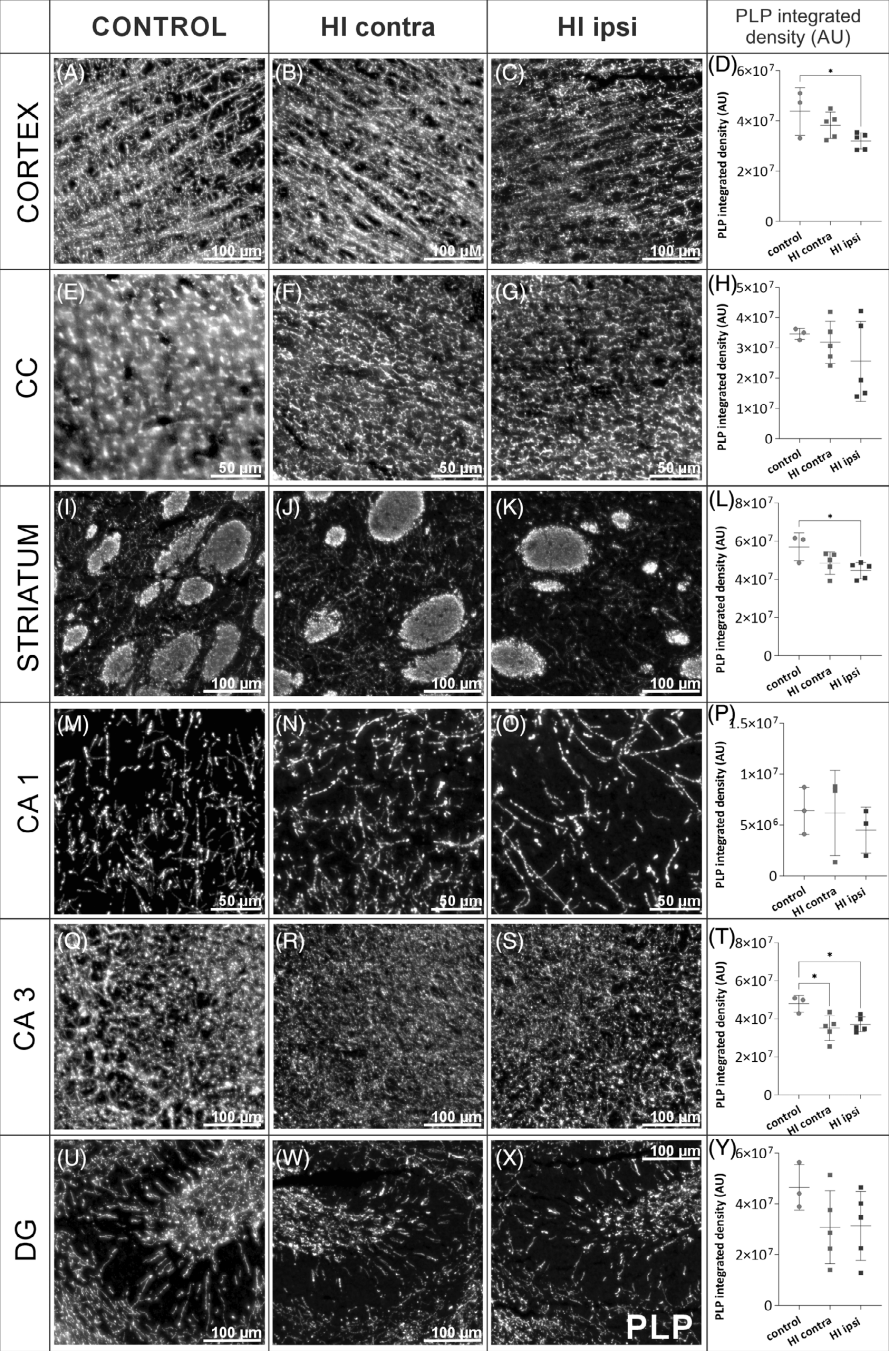
Results of myelination analysis with fluorescent microscopy 10 weeks after HI. Slices for the microscopic evaluation of brain myelination were collected from animals 10 weeks after HI (control *N* = 3; post‐HI *N* = 5) and stained with anti‐PLP (white). The figure contains example regions of interest (ROIs) from PLP‐stained slices from which the mean fluorescence intensity was measured (each brain region with 1–4 ROIs analyzed). Measurements were performed in ImageJ software and they are presented as the “integrated density” parameter in arbitrary units (AU). Brain regions analyzed included: cortex (A–D), corpus callosum (CC) (E–H), striatum (I–L), cornu ammonis 1 (CA1) of the hippocampus (M–P), cornu ammonis 3 (CA3) of the hippocampus (Q–T), and dentate gyrus (DG) of the hippocampus (U–Y). Scale bar on microscopic images corresponds to 50 μm or 100 μm as indicated on each image. Normal distribution was tested with the Shapiro–Wilk test. Statistical significance of the obtained data with normal distribution was evaluated by one‐way ANOVA test with Tukey's correction. Statistically significant differences. HI, hypoxia–ischemia. *p < 0.05.

In order to evaluate myelination at the microstructural level, an immunofluorescence staining with anti‐PLP in different frontal sections was performed. Figure 3 shows exemplary cross sections of the whole control brain and after HI injury 10 weeks after HI. In the HI ipsi hemisphere, similar to MRI results, there was observed an inhibition of striatum development after injury (Figure 3A,B), decreased thickness of the corpus callosum (Figure 3C,D **)**, and hypotrophy of hippocampal formation (Figure 3E,F). The decrease in the white matter and/or myelin can result neuronal tissue loss as well as an inhibition of oligodendrocyte differentiation, or oligodendrocyte lineage cell death after the injury. These processes were not investigated in the current work. However, as we did not observe significant focal damage, our focus was to assess whether even mild HI damage could induce white matter alterations. A more accurate assessment of changes in the myelination of selected brain regions was possible by measuring PLP fluorescence of selected brain regions. We used an “Integrated Density” parameter in ImageJ software, which calculates the product of the selected area and the mean grey value to normalize the results of the fluorescence measurements. Within the cortex, the amount of PLP protein was reduced by one‐third in the HI ipsi hemisphere compared to CT (Figure 4A–D; *p* < 0.05). Performed analyses did not reveal statistically significant changes between control and injured tissue in the region of the corpus callosum, the main white matter tract, although there was a trend toward reduced immunoreactivity (*p* = 0.42; Figure 4E–H). Nerve fibers in the striatum contained on average 21.8% less PLP in HI ipsi hemisphere (Figure 4I–L; *p* < 0.05). HI did not induce a significant effect on the amount of PLP myelin protein produced in the CA1 and DG regions of the hippocampus (Figure 4M–P,U–Y). Interestingly, in the CA3 region of the hippocampus, the most myelinated hippocampal area, 22.5% lower PLP immunoreactivity was measured in the HI ipsi hemisphere compared to CT (Figure 4Q–T).

#### 3.1.3 Myelination after neonatal HI evaluated with TEM

**FIGURE 5:**
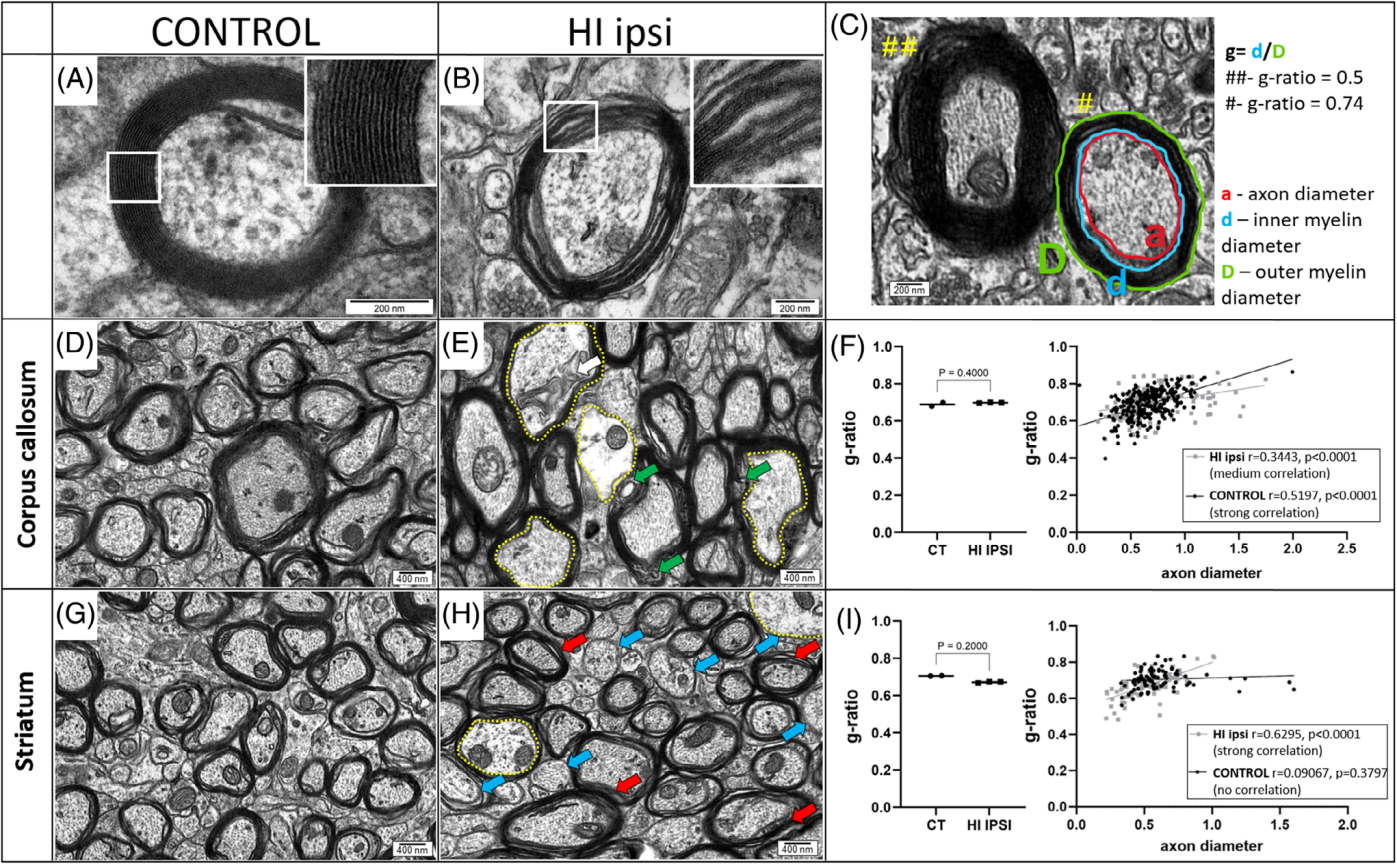
Results of myelination analysis with TEM 9 weeks after HI. TEM was used to assess the myelination at ultrastructural level. Average myelin segment from control tissue is a multi‐layer, compact structure (A), while in HI tissue, there are more often observed separate layers of myelin wrapping (B). The g‐ratio parameter was used to measure the thickness of myelin sheaths and it was calculated according to the presented formula (C) with the MyelTracer software. Analyzed regions included CC and striatum (*N* = 5, each brain region with an average of 94 axons measured). Within the CC of control tissue (D) as well as HI ipsi (E) tissue, there are axons wrapped with compact as well as less compact myelin, but in injured tissue, there are numerous vacuolations of myelin sheath, indicated with green arrows. The yellow dotted line outlines the axons with signs of swelling and cytoskeletal disintegration, one of which has multilayered structures that may arise from the inner myelin (white arrow). Despite the presence of numerous abnormalities in the structure of myelin sheaths, there is no difference in g‐ratio between control and HI ipsi tissue of CC (F). Control striatum with its normal myelination is presented in image (G), whereas in HI ipsi tissue, there is an increased number of uncompact myelin indicated by red arrows (H). Moreover, there are more non‐myelinated axons, as indicated with blue arrows. Results of obtained g‐ratio measuring were tested for normal distribution with the Shapiro–Wilk test. Statistical significance of the obtained results was evaluated by unpaired two‐tailed Mann–Whitney test. Correlation between axon diameter and g‐ratio was evaluated with Pearson's correlation test (F–I). CC, corpus callosum; HI, hypoxia–ischemia; TEM, transmission electron microscopy.

Deeper insight into the structure of myelin sheaths within the major white matter tracts was possible with the TEM technique. Myelination was evaluated in the electronograms collected from corpus callosum and striatum of control and HI ipsi hemispheres 9 weeks after HI. The overall evaluation on myelin structure revealed lower compaction of myelin around axons in HI ipsi tissue (Figure 5A,B). To evaluate the efficiency of myelination by means of myelin thickness, we measured the diameters of inner and outer myelin and calculated g‐ratio (Figure 5C). Numerous myelin vacuolations were observed within corpus callosum, both in control and HI tissue (Figure 5D,E). As these may arise as artifacts during tissue processing, their nature can only be indicated by the slightly different structures in control and damaged tissue. In this structure, we did not observe differences in measured g‐ratio (Figure 5F). However, the massive, persistent damage to the nerve tissue is indicated also by numerous axons with signs of swelling and cytoskeletal disintegration (Figure 5E). Less overall damage of myelin was observed within striatum, as well as g‐ratio did not significantly change after HI (Figure 5G–I).

#### 3.1.4 Oligodendrocyte proliferation after neonatal HI

**FIGURE 6:**
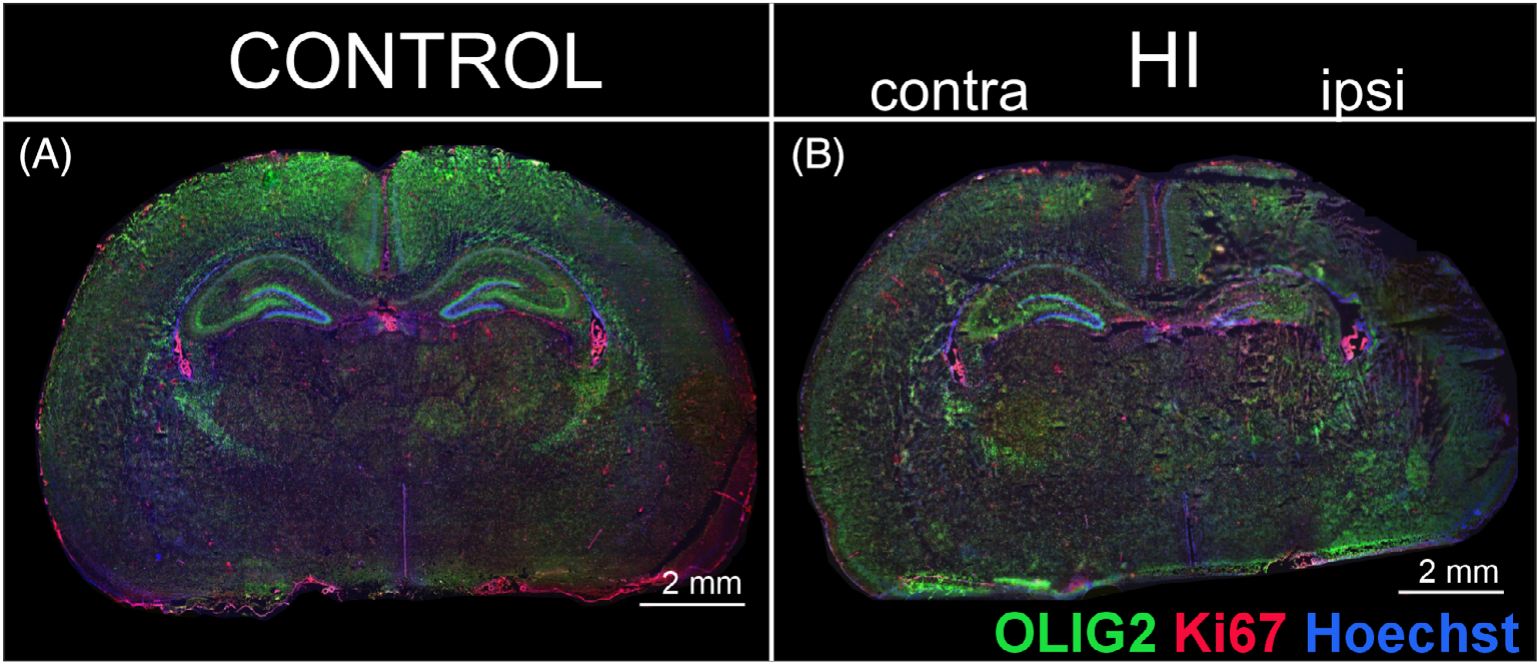
Overview image of brain tissue damage 3 days after HI. Microscopic images of whole brains in frontal section covering hippocampus, fixed 3 days after HI, stained with anti‐OLIG2 (green), anti‐Ki67 (red), and Hoechst (blue). In control slice (A), individual brain regions are well differentiated with the staining used. Although there was no specific test performed to assess the tissue damage, the overall picture of the injury is clearly visible in HI tissue (B), especially in the HI ipsi hemisphere. HI, hypoxia–ischemia. Scale bar corresponds to 2 mm.

**FIGURE 7:**
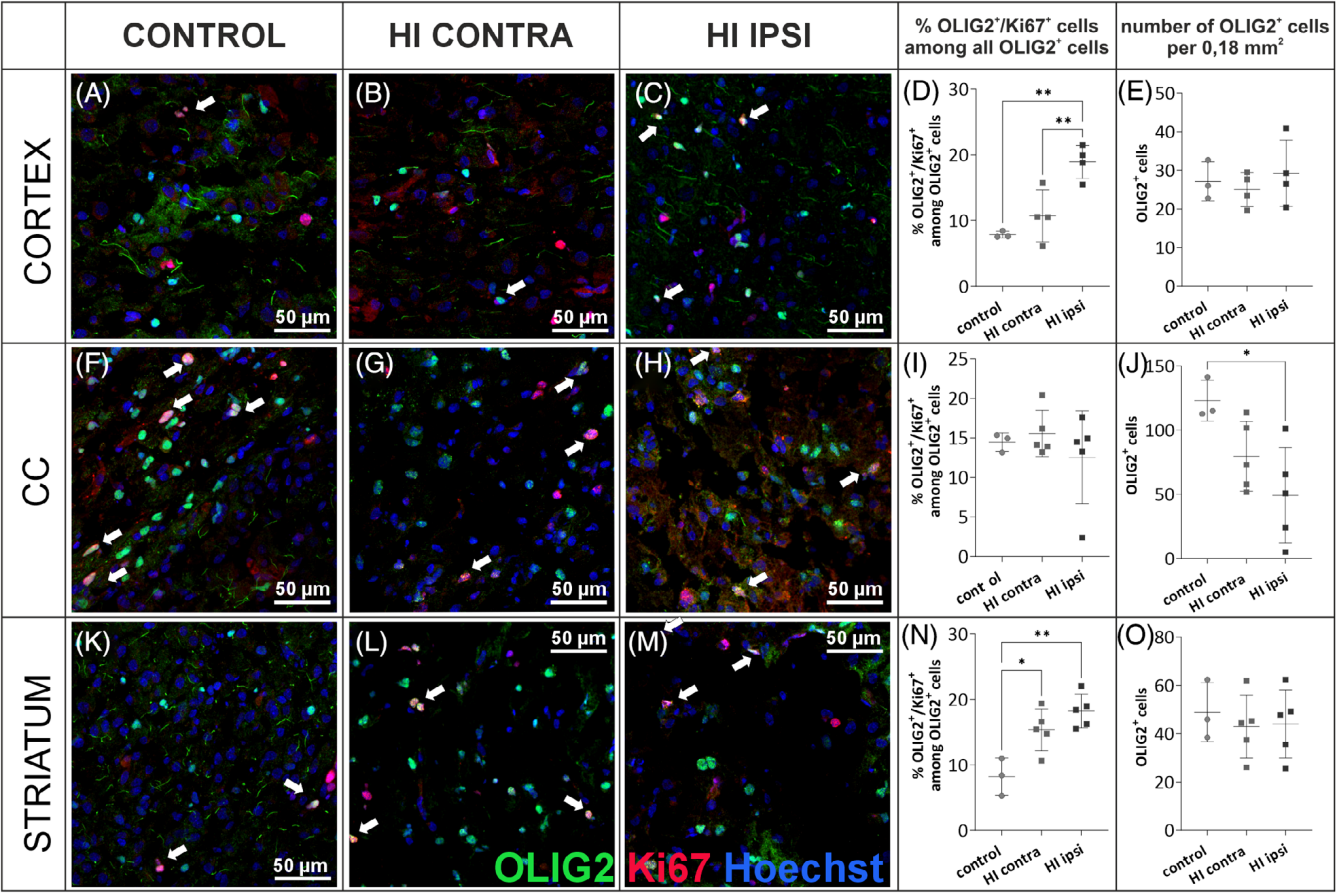
Results of the evaluation of oligodendrocyte distribution and proliferation 3 days after HI in cortex, CC, and striatum. Representative images of brain sections from control (*N* = 3) and post‐HI (*N* = 5) animals and results of counting positively labeled cells in slices fixed 3 days after damage induction and stained with anti‐OLIG2 (green), anti‐Ki67 (red), and Hoechst (blue). The regions analyzed included: cortex (A–E), CC (F–J), and striatum (K–O); each brain region with 16–48 images analyzed. Normal distribution was tested with the Shapiro–Wilk test. Statistical significance of the obtained data with normal distribution was evaluated by one‐way ANOVA test with Tukey's correction. CC, corpus callosum; HI, hypoxia–ischemia. *p < 0.05; **p < 0.01. Scale bar corresponds to 50 μm.

**FIGURE 8:**
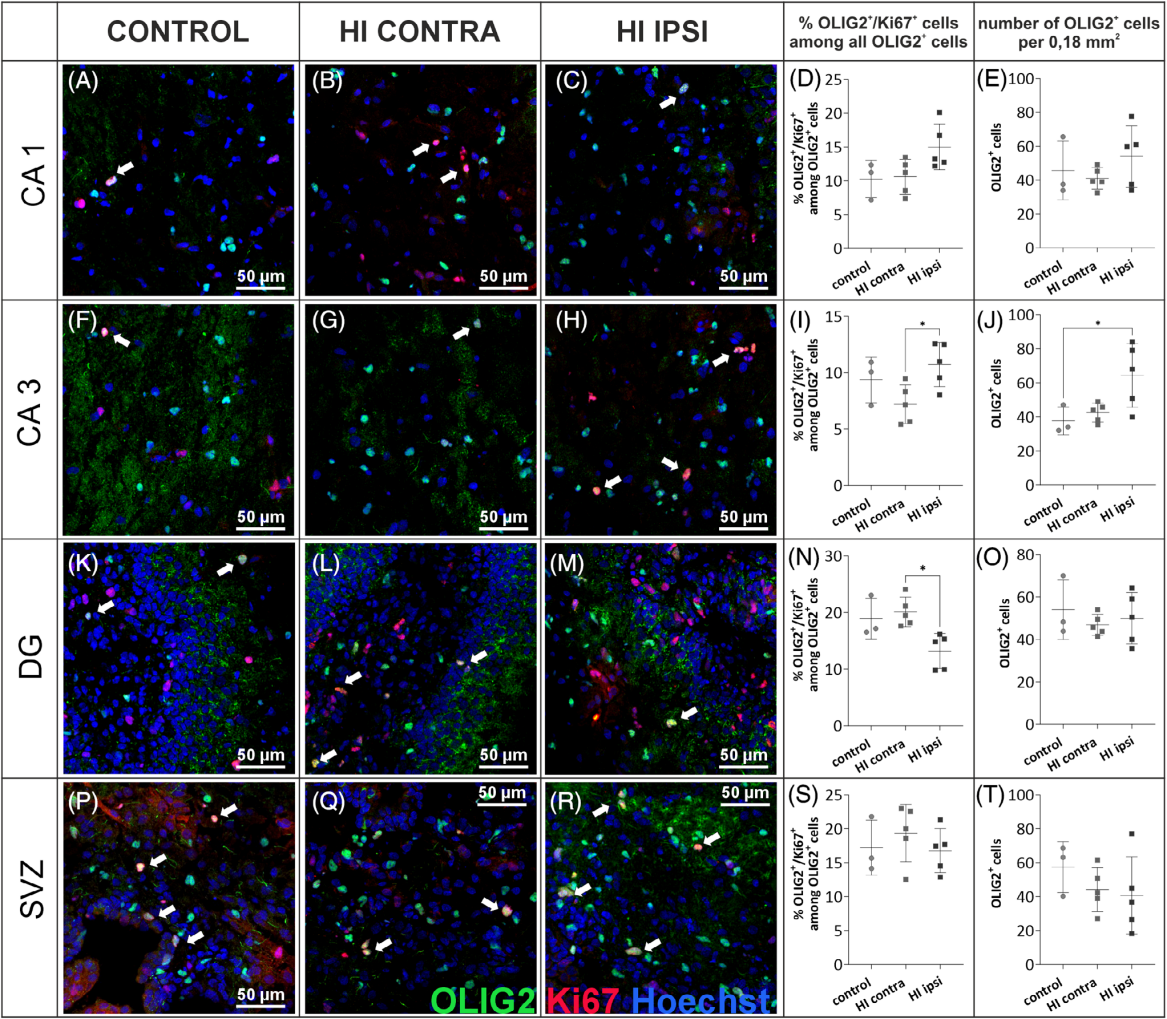
Results of the evaluation of oligodendrocyte distribution and proliferation 3 days after HI in CA1, CA3, and DG of the hippocampus. Representative images of brain sections from control (*N* = 3) and post‐HI (*N* = 5) animals and results of counting positively labeled cells in slices fixed 3 days after damage induction and stained with anti‐OLIG2 (green), anti‐Ki67 (red), and Hoechst (blue). The regions analyzed included: CA1 of the hippocampus (A–E), CA3 of the hippocampus (F–J), DG of the hippocampus (K–O), and subventricular zone (P–T); each brain region with 16–48 images analyzed. Normal distribution was tested with the Shapiro–Wilk test. Statistical significance of the obtained data with normal distribution was evaluated by one‐way ANOVA test with Tukey's correction. CA, cornu ammonis; DG, dentate gyrus; HI, hypoxia–ischemia. *p < 0.05; **p < 0.01. Scale bar corresponds to 50 μm.

To assess the fate of oligodendrocytes shortly after the injury, whether there is a loss of these cells or impaired maturation, microscopic analyses of tissues obtained from P10 animals (3 days after HI) were performed. Coronal brain sections were stained with anti‐OLIG2 and anti‐Ki67 markers. This allowed the assessment of changes in the number of oligodendrocyte linage cells (OLIG2+) as well as proliferating OPCs (OLIG2+/Ki67+) even before myelin changes could be observed. Imaging of immunolabeled whole brain slices indicates significant tissue damage after HI, particularly within the HI ipsi hemisphere. There is also an altered distribution of cells labeled with the anti‐OLIG2 marker (green) in various regions of the brain, mainly within the cortex and the corpus callosum (Figure 6). To further evaluate the density of oligodendrocytes and their proliferation in different brain regions, a series of microscopic images of areas 0.18 mm^2^ in size were taken, in which the number of cells expressing OLIG2 within the cell nucleus and cells expressing the proliferation marker Ki67 was counted. Representative images corresponding to the areas examined are shown in Figures 7 and 8. The brain regions with intensive production of myelin sheaths, such as cerebral cortex and, later in development, myelinated nerve fiber clusters of the corpus callosum and striatum, in normal development are characterized by the presence of maturing cells, thus with lower proliferation capacity. Meanwhile, after the onset of HI, a significant increase in OLIG2^+^ cell proliferation was observed in the cortex and striatum region. In the cortex, an average of 7.86 ± 0.51% of OLIG2^+^ cells expressed Ki67 in control tissue, while 18.91 ± 2.52% of cells expressed Ki67 in HI ipsilateral hemisphere (*p* < 0.001; Figure 7A–E), what was an increase of 240.6%. Similarly, more than a twofold increase in OLIG2^+^ cell proliferation was observed in the striatal region (8.21 ± 2.85% in CT; 18.24 ± 2.56% OLIG2^+^/Ki67^+^ cells in HI ipsi; *p* < 0.001; Figure 7K–O). This may indicate an expansion of OPCs as well as inhibited maturation of oligodendrocytes. Here, we also observed a significant increase in cell proliferation in the HI contra hemisphere compared to CT, induced only by periodic oxygen limitation (*p* < 0.01). No differences in the proliferation of oligodendrocyte lineage cells were observed in corpus callosum. In contrast, the total number of OLIG2^+^ cells decreased in this region, especially in the HI ipsi hemisphere (*p* < 0.05; Figure 7F–J). Another brain structure particularly affected by HI injury is the hippocampal formation. Interestingly, despite no effect of HI on OLIG2^+^ cell proliferation and the overall number of OLIG2+ cells in the CA1 region of the hippocampus (Figure 8A–E), CA3 showed an increase in proliferation and number of oligodendrocytes after HI. In the HI ipsi relative to HI contra, the number of proliferating OLIG2+ cells increased by an average of 3.5 (*p* < 0.05) and the overall number of OLIG2^+^ cells increased by an average of 26.7 compared to control tissue (*p* < 0.05; Figure 8F–J). The DG of the hippocampus is the site of intense proliferation of progenitor cells. It appears, however, that although the total number of OLIG2^+^ cells in this region does not change under the influence of injury, a 34.3% (*p* < 0.05) reduction in the proliferative capacity of these cells was observed in the HI ipsi hemisphere compared to the HI contra hemisphere (control 18.93 ± 3.59%, HI contra 20.11% ± 2.6%; HI ipsi 13.21% ± 3.08% OLIG2^+^/Ki67^+^ cells; Figure 8K–O) which may indicate an accelerated, premature differentiation effect or migration of oligodendrocyte progenitors to other sites of the brain. Additionally, proliferation analysis was performed in the SVZ (subventricular zone), which is not a myelinated area but also represents a site of intense progenitor cell proliferation. In this region, the proliferation of OLIG2^+^ cells as well as their overall number were not affected (Figure 8P–T).

#### 3.1.5 Myelin protein synthesis after neonatal HI

**FIGURE 9:**
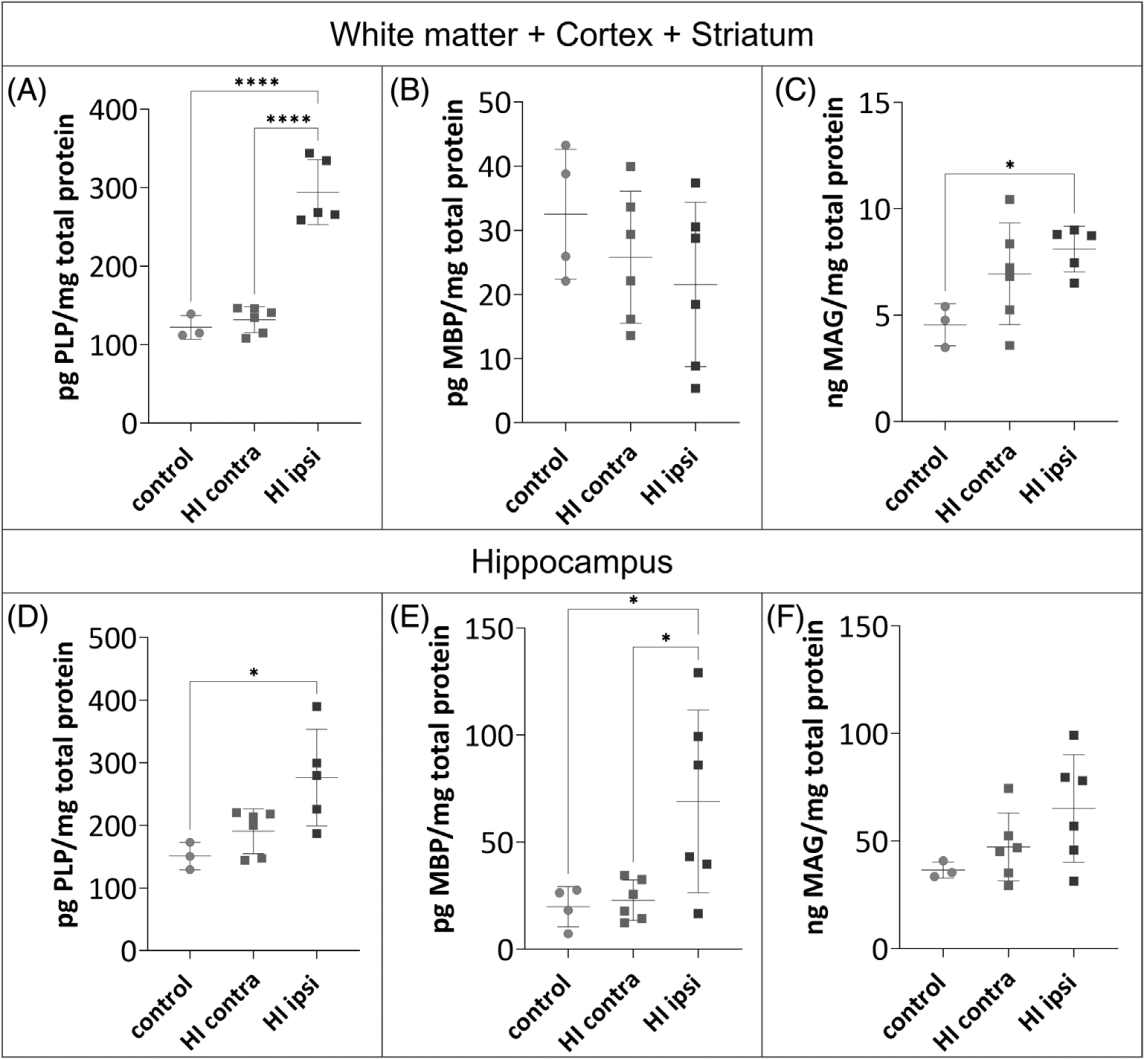
Results of the myelin proteins concentration in tissue. To evaluate the changes in myelin proteins: PLP (A, D), MBP (B, E), and MAG (C, F) in homogenates of control (*N* = 4) and post‐HI (*N* = 6) tissues collected 4 weeks after injury induction, the ELISA was used. Results were normalized to the concentration of total protein in the sample measured with DC Protein Assay. Normal distribution was tested with the Shapiro–Wilk test. Statistical significance of the obtained data with normal distribution was evaluated by one‐way ANOVA test with Tukey's correction (A–C, E,F). Statistical significance of the obtained data without normal distribution (D) was assessed by the Kruskal–Wallis test with Dunn's correction. HI, hypoxia–ischemia. *p < 0.05; ****p < 0.0001.

In order to discriminate, whether the change in proliferation concomitant with an opposite effect in the number of OLIG2^+^ may indicate premature or inhibited oligodendrocyte maturation, we evaluated the amounts of MBP, PLP, and MAG 4 weeks after HI, when myelin production reaches its maximum in adolescent rats (around P35; [49]). The hippocampi were isolated from the brain hemispheres and thus two types of samples were obtained: a hippocampal and hemispheres lacking the hippocampus (white matter, cortices, and striatum). The results indicate an increased expression of almost all proteins tested in the HI ipsi hemisphere (Figure 9). The amount of PLP protein in the HI ipsi increased by 1.8‐fold and by 2.4‐fold compared to the HI contra hemisphere, in hippocampal and non‐hippocampal brain hemisphere samples, respectively (*p < 0.0001*; Figure 9A,D). Hypoxic damage did not affect MBP protein expression: the amount of this protein was comparable in CT and HI contra hemispheres (Figure 9B). In hippocampal samples, HI damage caused a significant increase in MBP protein relative to CT and HI contra hemisphere samples (*p* < 0.05; Figure 9E). Among the proteins analyzed, MAG is present at relatively the lowest concentration. This protein is present only during myelination of the axon by the oligodendrocyte. However, after damage had occurred, MAG was overexpressed in samples from brain hemispheres without hippocampus within the HI ipsi hemisphere compared to CT (8.1 ± 1.07 pg MAG/mg total protein vs. 4.55 ± 0.98 pg MAG/mg total protein, respectively; *p* < 0.051). Despite a different effect of HI on OLIG2^+^ cell proliferation in both analyzed sites (increase in cortex and striatum and decrease or mild effect in hippocampus), overproduction of myelin proteins prevails in both analyzed regions late after damage. Since these results did not give a clear answer, a study was conducted on a complementary in vitro model.

### 3.2 Evaluation of the effect of neonatal HI on oligodendrocytes in the in vitro model

#### 3.2.1 OPC viability after OGD

**FIGURE 10:**
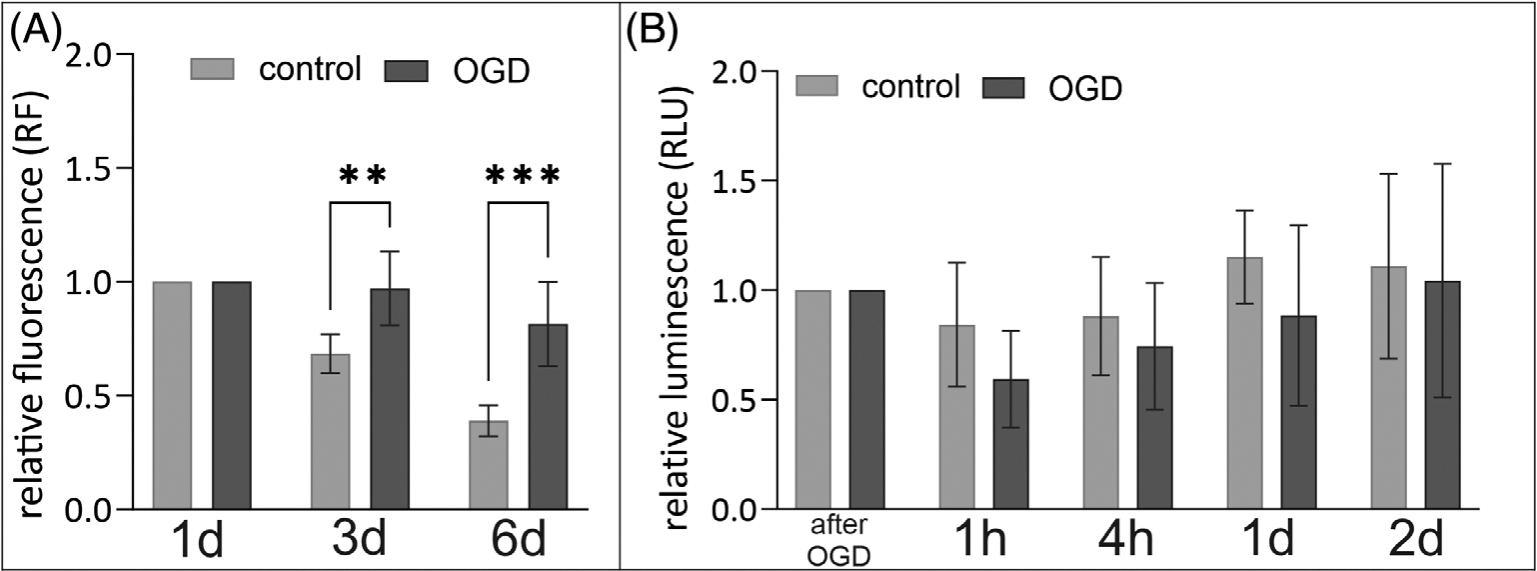
Evaluation of cell viability in the in vitro OPC cultures treated with OGD. (A) Results of the alamarBlue test at 1, 3, and 6 days after OGD. (B) Results of LDH assay of OPCs culture immediately after OGD, after 1 h, 4 h, and 1 and 2 days. Obtained data were normalized with values obtained in the first analyzed time point. Normal distribution was tested with the Shapiro–Wilk test. Statistical significance of the obtained results was evaluated by multiple Mann–Whitney tests. LDH, lactate dehydrogenase; OGD, oxygen‐glucose deprivation; OPC, oligodendrocyte progenitor cell. **p < 0.01; ****p < 0.0001.

To explain the lack of consistency between increased proliferation and production of myelin proteins and myelination after injury in adolescent animals, the procedure was recapitulated in vitro on isolated rat OPCs. Primary neonatal rat OPC cultures were subjected to OGD 4 h after cell seeding. The viability of OPCs cultures after OGD was assessed by alamarBlue assay. The obtained fluorescence intensity results were normalized with respect to the number of seeded cells per well of the 24‐well plate and fluorescence obtained in the first time point of analysis. In this way, values of the so‐called Relative Fluorescence (RF) were obtained. In supernatants collected from OPC cultures at Days 3 and 6 after OGD, significantly higher fluorescence was observed relative to control cultures. Thus, higher fluorescence values are associated with increased metabolic activity of cells or with an increase in the number of cells in culture after OGD (Figure 10A). To verify the result of the alamarBlue assay, an additional assay was performed based on the measurement of LDH enzyme activity secreted by the dead cells into the culture medium. The results obtained confirmed the results obtained earlier. No increased dying of OPCs after OGD was observed in in vitro culture within 2 days after OGD (Figure 10B).

#### 3.2.2 OPC proliferation after OGD

**FIGURE 11:**
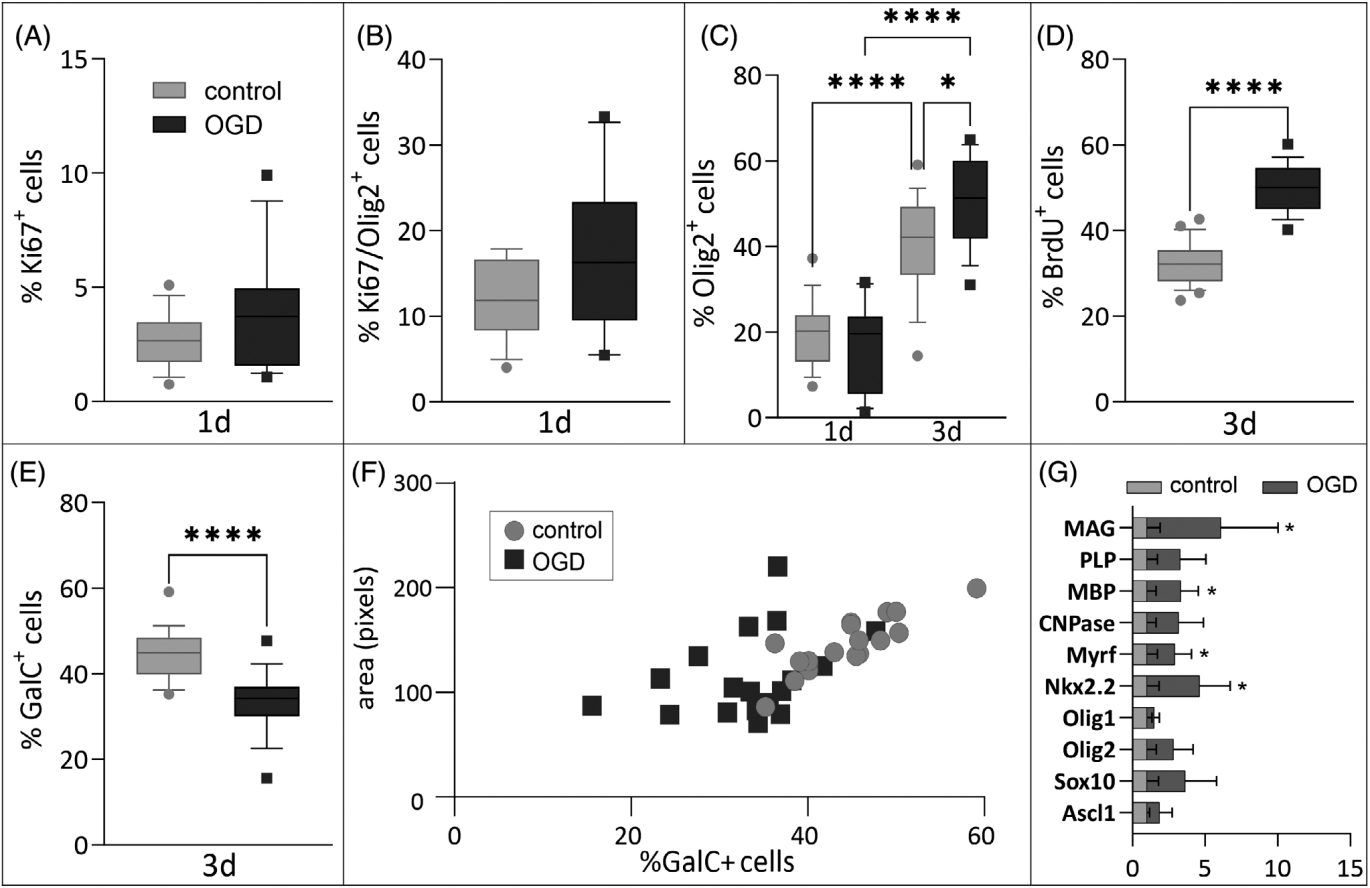
Evaluation of OPC proliferation and maturation after OGD in vitro. (A) The percentage of Ki67 expressing proliferating cells among all Hoechst^+^ cells 1 day after OGD. (B) The percentage of Ki67^+^ cells among cells expressing OLIG2 1 day after OGD. (C) The percentage of OLIG2^+^ cells among all Hoechst^+^ cells 1 day and 3 days after OGD. (D) The percentage of BrdU^+^ cells among cells expressing PDGFαR 3 days after OGD. (E) The percentage of GalC^+^ cells among all Hoechst^+^ cells 3 days after OGD. (F) Pearson's correlation analysis between the number of GalC^+^ and the area covered by these cells (control: *r* = 0.8458, *p* < 0.0001; OGD: *r* = 0.3483, *p* = 0.1567). (G) Results of the expression of genes encoding transcription factors involved in the differentiation of early neural progenitors into oligodendrocytes (Ascl1, Sox10, and Olig2), transcription factors involved in the maturation of oligodendrocytes (Olig1, Nkx2.2, and Myrf) and myelin‐specific proteins (Cnp, Mbp, Plp, and Mag). Isolation of mRNA performed on Day 1 after OGD. Results were normalized against Rpl13 and B2m expression. Normal distribution was tested with the Shapiro–Wilk test. Statistical significance of the obtained data with normal distribution was evaluated by unpaired two‐tailed *T* test (A,B, D,E, G); two‐way ANOVA test with Tukey's correction (C) or Pearson's correlation (F). OGD, oxygen‐glucose deprivation; OPC, oligodendrocyte progenitor cell. *p < 0.05; ****p < 0.0001.

Results of viability assays may indicate that OGD might promote OPC proliferation. This was verified with the immunofluorescence labeling of cells, with Ki67 marker of proliferation. It enabled visualization of cells undergoing cell division at the time of fixation of the cells. The results obtained did not indicate statistically significant differences from controls (Figure 11A,B); however, the tendency for enhanced Ki67 expression allows us to presume that the short‐term deprivation of oxygen and glucose leads to increased cellular proliferation immediately after injury. This effect was confirmed with an increased total number of OLIG2^+^ cells in culture 3 days after OGD (mean in control 12.17 ± 1.29% vs. 17.01 ± 2.83% in OGD; *p* < 0.05; Figure 11C) as well as BrdU incorporation test. The analysis performed 3 days after OGD revealed a 55% increase in the number of newly formed cells co‐labeled with PDGFRα (*p* < 0.0001; Figure 11D).

#### 3.2.3 OPC differentiation after OGD

**FIGURE 12:**
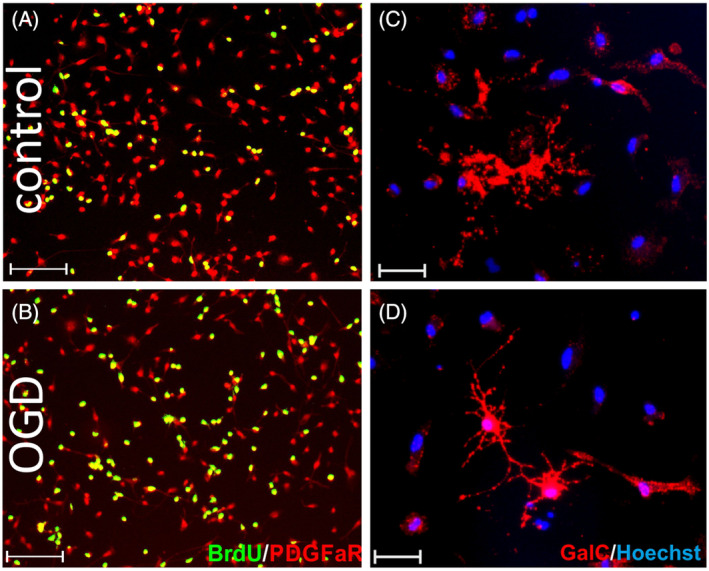
Representative microscopic images from the evaluation of OPC proliferation and maturation after OGD in vitro. Control (A) and OGD‐treated (B) cultures stained with anti‐PDGFaR progenitor cell marker (red) and anti‐BrdU (green). Control (C) and OGD‐treated (D) cultures stained with anti‐GalC (red). Cell nuclei stained with Hoechst (blue). Scale bar corresponds to 100 μm. OGD, oxygen‐glucose deprivation; OPC, oligodendrocyte progenitor cell.

There was a reduction in the number of immature and mature oligodendrocytes with GalC (galactocerebroside) expression at Day 3 after OGD (from 44.23 ± 5.92% in controls to 33.28 ± 7.24% in OGD‐treated cultures; *p* < 0.0001; Figure 11E). A Pearson correlation coefficient was calculated to assess the relationship between the percentage of GalC^+^ cells and the area of the culture vessel they cover (Figure 11F). There was a strong, positive, and significant correlation between the two variables in control cultures (*r* = 0.8458; *p* < 0.0001), while no such correlation was observed in OGD cultures (*r* = 0.3483; *p* = 0.1567). This may indicate that GalC^+^ cells after OGD, even though they express oligodendrocyte proteins, are less able to produce cell extensions and have smaller sizes. This assumption was verified with the quantitative analyses of selected mRNA expression of transcription factors associated with oligodendrogenesis and myelin proteins at Days 1 and 3 after OGD. On the first day after OGD, we observed a more than threefold increase in the expression of two of the six analyzed transcription factors—Nkx2.2 and Myrf and also genes coding myelin proteins—Mbp and Mag (*p* < 0.05; Figure 11G). Three days after injury, the expression of all analyzed proteins remained at similar levels (*p* > 0.05; data not shown). Figure 12 shows representative microscopic images of analyzed PDGFRα/BrdU cells (Figure 12A,B) and GalC^+^ cells, where less complex morphology of OGD‐treated cells is observed (Figure 12C,D).

## 4 DISCUSSION

Perinatal HI remains a significant problem in neonatology and pediatrics. Although there are numerous reports published to date indicating possible mechanisms involved in the pathophysiology of developing white matter injury, the majority of them focus on the neuronal injury and death. It is becoming increasingly common to claim that oligodendrocytes should be the potential therapeutic target, as they are particularly affected, especially immature oligodendrocytes, which are predominant oligodendrocyte‐lineage cells in neural tissue during the neonatal period [50, 51, 52]. The only therapeutic strategy available, which is mild hypothermia, is based on the principle of “damage reduction,” that is, inhibiting further development of the inflammatory response and other mechanisms that contribute to cell apoptosis. Xiong et al. also demonstrated the potential contribution of therapeutic hypothermia in increasing oligodendrocyte survival and promoting their differentiation [51]. However, it is unclear how perinatal asphyxia affects oligodendrocyte cells, which are at an early stage of differentiation during a HI episode. Back et al. [26] showed that OPCs are relatively insensitive to HI‐induced damage. However, it is not fully understood how HI damage may influence the subsequent oligodendrocyte differentiation and if it affects this process directly.

In order to develop and test relevant therapeutic strategies for the white matter injury resulting from neonatal asphyxia it is particularly important to design and use the most relevant rodent model. The key is the age of the pups which implicates the developmental state of the brain. Applying the same HI procedure to rodents at different ages will result in different outcomes. Recent studies indicate that the effect of asphyxia in preterm babies is reflected in the HI injury induced in P7 rats, which were used in this study [53, 54].

Few groups in the field have so far evaluated the effect of HI on white matter development, especially with the MRI method. White matter development and injury can be evaluated with DTI analysis, which measures the directional diffusivity of water. A decrease in FA within the white matter regions was observed already at 1 day and 7 days after the procedure by Wang and colleagues [55], as well as at later time points by Widerøe et al. [56]. In another study, with the injury induced in P3 rats, reduction in FA was also observed 1 day after the injury in cortical layers, indicating alterations in their microstructure [57]. Until now, there was no such study presenting changes in FA obtained from P7 rat model of neonatal HI obtained from the same animal at different time points. We did not directly compare FA parameter between CT and HI groups, but evaluated how it changes in time. While FA increases significantly in time in selected regions of CT brains, especially main white matter tracts, these changes are not observed in HI ipsi tissue. Moreover, with the results of this study there also arose some more interesting questions – what is behind the increased FA in HI tissue in selected regions, like optic pathways compared to CT hemisphere, as well as a decrease in FA in other regions (values close to −1, red), both in control and HI animals. One hypothesis is that it may not be associated with the changes in myelination, but fiber density and directional organization changes during brain development [58]. Despite the fact that FA can be modulated by the content of myelin sheaths [59], and during the development of rat corpus callosum, the myelin sheath's area give the strongest contribution to FA value [60], there are methods that are more specified to evaluate myelination, especially in corpus callosum. Recent studies developed the measurements of myelin water [61] or g‐ratio measured with MRI [62] in humans, but such protocols were not yet reported in the rat model of neonatal HI. Nevertheless, thanks to the comprehensive study of different diffusivity parameters in rat brain development provided by Bockhorst et al. [63], we learned more about the normal brain development. Additionally, Lariosa‐Willingham et al. recently showed that testing promyelinating compounds can be evaluated with MRI imaging [64]. The other issue we were intrigued by was the difference in volumes of selected structures between the right and left hemispheres in control animals. Moreover, these differences disappeared after the HI injury. The asymmetry of brain hemispheres in humans and rodents, at functional and structural levels, is well documented [65, 66]. Interestingly, Postema et al. recently reported that in the case of autism spectrum disorders in humans, this asymmetry if often reduced [67]. Probably, an affected brain lateralization resulting from the injury, together with reduced volumes of hippocampus and somatosensory cortex observed in our model as well as myelination and white matter development deficits, may be involved in behavioral deficits of animals after HI [36].

In this study, we verified observations from MRI with microscopic imaging techniques which give deeper insight into the structure of the tissue. We visualized myelin with anti‐PLP immunofluorescence labeling. PLP is a transmembrane protein, is involved in the adhesion of myelin membrane layers during sheath formation, and accounts for nearly half of all myelin membrane proteins [68]. Macrostructure overview of the slices immunolabeled with anti‐PLP indicated in the HI ipsi hemisphere, a reduced striatum, decreased thickness of the corpus callosum, and hypotrophy of hippocampal formation. These observations were similar to MRI volumetry measurements. Such features could also develop in preterm babies with asphyxia [9]. Measuring PLP fluorescence intensity revealed myelination deficits in the cortex, striatum, and CA3 region, which under physiological conditions has the highest expression of myelin proteins in the entire hippocampal formation [69]. Interestingly, a simple comparison of this result with presented FA changes over time measured by MRI may not be conclusive. However, comparing average absolute FA values from similar time points between treated groups reveals similar observations (Supporting Information: Figure 1), with tendency to reduction in FA value, reflecting reduced myelination in most of the analyzed brain regions. One exception was striatum, where FA decreased only in contralateral HI hemisphere compared to both the control and HI ipsi hemispheres. That observation also confirms that FA cannot be used to directly evaluate myelination, since it relies not only on myelin content, but also an axonal membranes which can affect the diffusion process [59, 70].

TEM is a gold standard for investigating the morphology of cells and the processes in which they are involved. It enables determining the condition of brain tissue at different stages of neuroregeneration after HI, for example, observation of apoptosis of neural cells within a few days after the injury, where compaction of chromatin is typical pattern [71, 72]. The effect of HI in rodent models of HI was already evaluated by different groups, but usually they observed increased g‐ratio, which means—thinner myelin in HI axons already at 3 weeks after HI [73, 74], as well as later in development [75]. Ueda et al. noticed no changes in the myelin ultrastructure [76] and Skoff et al. reported increased number of unmyelinated axons in the striatum [77], which was consistent with our results. Detailed analysis of myelin ultrastructure in young adult rats after HI has not been previously reported. Here we can also compare them to tissue analyses performed with different techniques. In accordance with the fact that no changes were observed in the PLP immunoreactivity after HI damage, the g‐ratio in CC was also not affected. But, besides measuring g‐ratio, we provided evidences for other myelin abnormalities which can affect its functionality in CC. Interestingly, changes in striatal myelin not observed with MRI but revealed with PLP immunostaining were confirmed with TEM where numerous non‐myelinated axons were observed.

With MRI method as well as ultrastructural analysis of brain slices, we revealed changes in the white matter's physical properties as well as myelin sheath deformation, which may impact its functionality. Eager to take a step back to see if the observed changes are actually caused by a reduced pool of oligodendrocyte cells producing myelin components, we evaluated the number of OLIG2^+^ cells 3 days after HI. Interestingly, we observed an increase in the number of OLIG2^+^ cells in CA3 of hippocampus and an increase in their proliferation in the cortex and striatum. The loss of OLIG2^+^ cells in the injured CC could possibly be later compensated to some extent caused by the migration of OPCs from SVZ [78], indicated by a tendency to decrease in the number of OLIG2^+^ cells observed in this region in our study and no changes in cell proliferation, as well as previously reported results on the BrdU‐labeled oligodendrocyte progenitors [47]. A similar view about the possible migration of OPCs from SVZ into regions of damage was also suggested by Gonzalez‐Perez and Alvarez‐Buylla [79].

This could be the case that HI did not cause massive oligodendrocyte loss, but increased their proliferation and/or affected their differentiation and maturation. Unfortunately, we did not analyze the viability of oligodendrocytes after the injury, but evaluated the level of myelin proteins PLP, MBP, and MAG that they produce. MBP is an important myelin membrane protein that interacts with its lipid components [80]. MAG is a glycoprotein that is produced only in myelinating oligodendrocytes and is responsible for myelin membrane interactions with neuronal axons. ELISA allowed us to quantitate these proteins in tissue lysates at the time point, when brain myelin production and brain growth reach its peak in adolescent rats, which is around 1 month of age [49, 81]. Surprisingly, with all analyzed proteins, we observed an increased concentration in most of the HI ipsi samples compared to controls. In 2008, Segovia et al. already noted that in a model of neonatal HI induced in P3 rats, there is an accumulation of oligodendrocytes at an early stage of maturation, which, however, are not capable of producing myelin sheaths and are susceptible to delayed apoptosis [82]. Similar observations have also been made in recent years in another model of perinatal encephalopathy caused by chronic inflammation in rats lasting from 1 to 3 days [83] or from 1 to 5 days after birth [84]. On the other hand, focusing only on CC, Lin et al. observed a decrease in MBP level at the same time point but from the injury‐induced 3‐day‐old rats [85], as well as Cai et al. who used a 4‐day‐old rat model of the injury, both modeling asphyxia affecting very preterm babies. Studies by Affeldt et al. in a mouse model of neonatal hypoxia (without induction of unilateral ischemia) showed reduced expression of the transcription factors Ascl1 and Olig1 7 days after injury and, consequently, inhibited production of the myelin proteins MBP and MAG [100]. In our model, with HI induced in P7 rats, the obtained effect might be different caused by different stages of oligodendrocyte and the entire neural tissue maturation. In the same paper, Lin et al. demonstrated that P4–P17 is the time when the number of OPCs and mature oligodendrocytes changes the most dynamically [85]. Moreover, Craig et al. demonstrated that between P3 and P7 oligodendrocyte lineage cells in the brain switch from pre‐OLs to immature oligodendrocytes being more prevalent [17], thus their vulnerability to the HI injury might be dramatically different. Numerous studies indicate that myelin components inhibit OPC differentiation [86, 87, 88] which is a serious problem in neurodegenerative disorders, mainly multiple sclerosis [89], limiting the process of endogenous myelin repair (remyelination). If this could be the case in our study, then the elevated concentration of myelin proteins and increase in the number of OLIG2^+^ shortly after the injury, may indicate premature oligodendrocyte differentiation.

In order to follow more deeply the fate of oligodendrocyte lineage cells we performed a complementary model but in the in vitro conditions. Here, we have shown by two independent methods (alamarBlue assay and LDH assay) that cells after OGD have increased metabolic activity, which may mean that their number in culture increases and/or their maturation is accelerated. Especially, the interpretation of the alamarBlue results was not necessarily straightforward. Indeed, as early as 1999, Back et al. indicated a positive correlation between the degree of cell differentiation and the metabolic activity detected by the alamarBlue assay. In the same study, the authors further demonstrated that the test result is not affected by factors that can stimulate oxidative stress, which is important in the context of the use of OGD [90]. However, the LDH assay results confirmed that OGD does not induce massive cell death. Among the papers published to date, we can find reports that only an OGD procedure lasting 120 min, which is three times longer than that used in the experiments performed in this study, can induce OPC death [91]. Wu et al. and Yanquin et al. even examined an OGD procedure lasting from 3 to 12 h and only oxygen deprivation lasting 9 h induced apoptosis of most cells in culture [92, 93]. The unique properties of OPCs can be evidenced by the fact that organotypic hippocampal slices, which were subjected to the same damage as OPCs in vitro, were characterized by lower viability in the alamarBlue assay (data not published). In this case, however, it should be considered that the results obtained may be affected by the presence of other dead cells, especially neurons, that are most sensitive to oxygen and glucose deprivation, which has been confirmed by numerous works performed on this model [94, 95].

The results of the alamarBlue viability assay described above may indeed indicate an increased proliferation of these cells after OGD. We proved this with immunofluorescence labeling. Similar results were obtained previously using the ex vivo model of HI injury with the procedure of OGD, where no difference in Ki67^+^ OPCs was observed 7 days after injury, but the number of BrdU‐labeled immature oligodendrocytes with O4 expression significantly increased [96]. A different effect of OGD on cell proliferation was observed by Yanqin et al. and Wu et al. [92, 93]. What is however worth notice, they cultured cells in media enriched with trophic factors.

The remaining questions, therefore, are whether newly formed progenitors after HI are capable of proper differentiation and whether local proliferation of these cells can, in effect, provide a new source of myelinating oligodendrocytes.

To assess the ability to differentiate, we measured the expression of genes encoding OLIG2 and other transcription factors involved in oligodendrocyte maturation. Similar studies have not been previously published, and the results obtained support the results from ELISA performed on samples from in vivo models. On Day 1 after OGD, the expression of Nkx2.2 and Myrf transcription factors active at the stage of myelin component production increased. Thus, the results obtained may imply accelerated differentiation of newly formed oligodendrocytes after OGD. Therefore, to verify this assumption, we assessed the presence of lipids characteristic of maturing oligodendrocytes and the expression of myelin proteins using immunofluorescence staining and RT‐qPCR. The cell membrane lipid detected early in oligodendrocytes is GalC. It is essential in the downstream stages of cell maturation and myelinization process for maintaining a stable structure of myelin sheaths [97]. The proportion of anti‐GalC‐labeled cells in in vitro culture on Day 3 after OGD was lower than under control conditions. Because of the small number of cells capable of producing myelinated membranes in in vitro culture, we did not perform immunofluorescence staining with markers specific for mature oligodendrocytes; instead, we analyzed myelin protein expression at the mRNA level. Changes for PLP and CNPase proteins were insignificant from the perspective of statistical analysis. However, in our previous studies with OGD performed on hippocampal slices, we showed that the injury limits the ramification of OPCs measured with Sholl analysis [24], but an increased MBP amount as well as the number of MBP^+^ oligodendrocytes can be observed with no significant changes in PLP concentration and reduced number of PLP^+^ cells [96]. Akundi et al., in their in vitro study, also observed that hypoxia (1% O_2_) promotes accelerated oligodendrocyte differentiation and significantly promotes MBP protein expression [98]. An analysis of gene expression for OLIG2 and MBP of cells after OGD was also previously performed by Ichinose et al. However, their observations showed that the procedure used has no effect on Olig2 expression but causes a decrease in Mbp expression 18 h after OGD [99]. Gathering all the findings from the experiments carried out in this study, we speculate that pathophysiological changes, especially those related to the disruption of ischemia‐induced cellular metabolism in neural tissue after HI, stimulate OPCs to mature and produce myelin proteins prematurely. Noteworthy are the observations obtained by histopathological examination of post‐mortem human tissues after diagnosed white matter damage in neonates, where Buser et al. demonstrated expansion of preOls, which may be inhibited from myelination by intense astrogliosis in the region of damage [100]. Besides the mechanical barrier for the myelination, astrocytes can also contribute to the increased proliferation of OPCs by secretion of numerous neurotrophic factors, for example, IGF‐1. We have recently shown that this factor is temporarily increased in neural tissue after HI and induces proliferation of OPCs when it is at high concentration [24]. There might be more questions and answers to find behind the phenomenon of impaired myelination followed by increased oligodendrocyte lineage cell proliferation and production of myelin proteins. Over the past few years, the view on which element in the whole scheme of cause and effect is the critical one, directly influencing the effects of proposed therapies, has been changing. Extensive multi‐center studies provide evidence for the important role of microglia cells and regulation of the inflammatory response [80, 101, 102, 103]. Many areas are still undiscovered, among them the role of HIF 1α‐regulated cellular processes after an HI incident that may stimulate premature overexpression of myelin proteins. Another interesting aspect may be the disruption of the autophagy process identified in apoptotic neural cells after ischemic injury [104, 105, 106]. Only recent reports indicate an important role of the process in the physiological maturation of oligodendrocytes, whereas the role in pathophysiological processes is still at the conjecture stage of researchers.

This study has however potential limitations. The main factor that may influence the evaluation of myelination and white matter development is the fact that it may be strongly affected by a potential loss of axons. This phenomenon was not assessed in the following study. However, our previous study documented a neuronal loss in the same model of HI injury [36, 46, 47, 48]. Axon loss was also observed in the chosen or similar models by other groups [107, 108, 109]. For this reason, we analyzed not only the total white matter volume, but also the myelin content normalized with the analyzed area in microscopy and the myelin ultrastructure in TEM. The fact, that loss of neural tissue was not estimated makes especially the interpretation of ELISA results not straightforward. An increase in the concentration of myelin proteins in HI ipsi hemisphere 4 weeks after HI may be an effect of an increased proliferation and overproduction of oligodendrocytes or—loss of axons to be myelinated. More studies with multiple markers of the oligodendrocyte lineage are necessary to verify this hypothesis. Noteworthy is the fact, that myelination of the surviving axons is impaired anyway, which is observed in TEM images. Although signs of myelination defect are evident in the ultrastructure of neural tissue, the statistical analysis of the results obtained is hampered caused by the small experimental groups in this experiment. The same issue was also a limitation in MRI. The cost of this experiment allowed us to imagine only five animals. Unfortunately the MR system at which the experiments were conducted does not offer sequences specific for myelination analysis, like myelin water imaging. Therefore, we decided to use FA as an estimator of myelination, as the area of myelin sheath has been reported to have the strongest contribution to the value of FA in the early stages of rat development [60]. Finally, we have not recorded the weights of animals or the weights of isolated brains. This would be a clear demonstration of the impact of HI damage not only on brain development, but on the whole organism. Studies from other research groups report however various observations on this parameter. No significant effect on body weight can be observed in the similar HI model of injury [110]; there may also be a mild effect depending on sex, with body weight reduction only in female animals with HI [111]. A significant effect on body weight reduction after HI was observed after prolonged hypoxia (120 min), where the contributing factor could be injury to the other organs, such as the kidney [6]. To conclude, the presented study provides new insights into the pathomechanism of oligodendrocyte differentiation and maturation failure after HI and describes in detail by means of diversified techniques the confirmed effects of HI on myelination of selected brain regions during neurodevelopment. The described in vitro and in vivo models provide valuable tools for further preclinical studies of compounds directed to improve white matter development and may enhance the translational potential of the obtained results.

## AUTHOR CONTRIBUTIONS

Justyna Janowska isolated OPCs, collected samples and performed biochemical analyses, confocal microscopy and analysed all the obtained data. Justyna Gargas established and cultured rat mixed glial cultures. Karolina Zajdel performed TEM imaging and Malgorzata Frontczak‐Baniewicz supervised TEM analyses. Michal Wieteska performed MRI imaging and, together with Kamil Lipinski, processed obtained MRI images. Malgorzata Ziemka‐Nalecz performed surgeries in the in vivo model. Justyna Janowska and Joanna Sypecka were awarded research funding. Justyna Janowska wrote the manuscript. Malgorzata Ziemka‐Nalecz and Joanna Sypecka contributed to the final version of the manuscript. All authors provided critical feedback and helped shape the analyses and manuscript.

## CONFLICT OF INTEREST STATEMENT

The authors declare no conflicts of interest.

## Supporting information


**Data S1.** Supporting Information.

## Data Availability

The data that support the findings of this study are available from the corresponding author upon reasonable request.
