# Supplementary material for: Oligodendrocyte progenitor cells' fate after neonatal asphyxia—Puzzling implications for the development of hypoxic–ischemic encephalopathy
Source: Brain Pathol. 2024 Mar 19;34(6):e13255. doi: 10.1111/bpa.13255 (PMC11483519; doi:10.1111/bpa.13255)
Supplement: Supplementary file 1 — Data S1. Supporting Information. [file BPA-34-e13255-s001.docx]

**Supplementary Information**

**Protocol 1. Immunofluorescence staining of fixed tissues and cells**

First step of immunofluorescent staining was blocking non-specific binding of antibodies. The composition of blocking solutions is listed in Supplementary Table 1. After 1 h of incubation in the blocking mixture at room temperature, the slides were washed 3 times with PBS solution and the primary antibody diluted in PBS was applied at different concentrations depending on the antibody used (Supplementary Table 1). After overnight incubation at 4°C, the slides were washed 3 times with PBS solution and the appropriate secondary antibodies diluted in PBS at 1:500 (tissues) or 1:1000 (cells) were applied for 1 h incubation at room temperature. When double staining was performed, the procedure was repeated using a second primary antibody from a different host. Stained slides were washed 3 times with PBS solution and cell nuclei labelling solution, Hoechst 33258 (Sigma-Aldrich; 1:150 in PBS), was applied for 15 min. The slides were then washed again with PBS solution and sealed with a coverslip or glass slide using Fluorescence Mounting Medium reagent (Dako).

**Protocol 2. Collecting and sampling microscopic images of immunofluorescence-labelled brain slices**

Measurement of PLP integrated density

The images of whole brain slices of anti-PLP labelled sections were collected with confocal microscope under magnification 20x. Images were automatically stitched with ZEN software (Zeiss). Images of selected brain regions (ROIs) of 0,16 mm2 (corpus callosum, hippocampal CA1 region) or 0,36 mm2 (cortex, striatum, hippocampal CA3 region, hippocampal DG region) were used for measurements. From each animal 1-3 different brain sections covering the same region were studied. Then, from each section, 1 to 3 ROIs were analyzed in each region. An image below presents the method of sampling ROIs for analysis. Myelination intensity of selected ROIs in microscopic images was measured using the "Integrated Density" function in ImageJ software, after cutting off the background fluorescence using the "Threshold" function. The "Integrated Density" value is calculated as the product of the area and the average fluorescence value. For each animal, region-specific averages were calculated and these data are shown in the graphs.


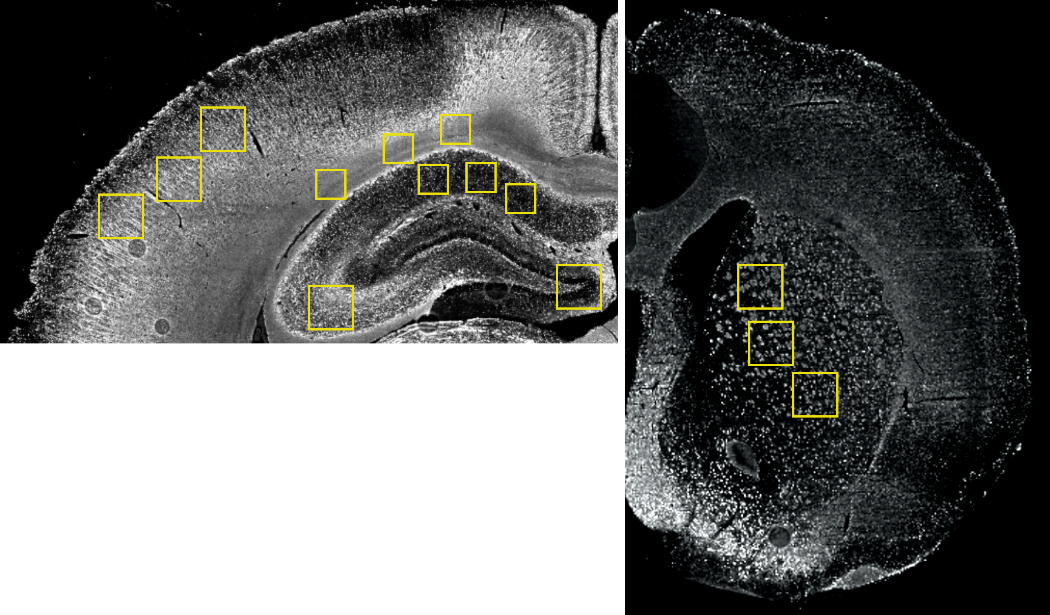


Counting of Olig2^+^ and Olig2/Ki67^+^ cells

The microscopic images of selected brain regions of Olig2/Ki67/Hoechst-labeled brain slices were collected with confocal microscope under magnification 20x. The collected images were 0,18 mm^2^ in size. Positively labelled cells were counted manually across the image using ImageJ software. Images were collected similar to the schematic image above. In addition, SVZ images were collected from striatum sections. From each animal 1-3 different brain sections covering the same region were studied. Then, from each section, 2 to 4 images were analyzed in each region. For each animal, region-specific averages were calculated and these data are shown in the graphs.

**Supplementary Table 1.** **List of reagents used for immunofluorescent microscopy.**

| Blocking mixture | **Brain slices**  PBS (Gibco)  Goat serum (Sigma) 10%  Triton X-100 (Serva) 0,25%  Bovine serum albumin (Sigma) 0,1% | | **Cultured cells**  PBS (Gibco)  Goat serum (Sigma) 10%  Triton X-100 (Serva) 0,1% |
| --- | --- | --- | --- |
| Antibodies |  | ***Primary*** | ***Secondary*** |
| Brain slices | **P10** | Rabbit IgG anti-**OLIG2** (1:500)  (MERCK; ABN899)    Mouse IgG1 anti-**Ki67** (1:100)  (Novocastra; NCL-L-Ki67-MM1) | Goat anti-rabbit IgG Alexa Fluor 488 (Invitrogen; A-11008)    Goat anti-mouse IgG1 Alexa Fluor 546 (Invitrogen; A-21123) |
|  | **P77** | Mouse IgG2a anti-**PLP** (1:100)  (Chemicon; MAB388) | Goat anti-mouse IgG2a Alexa Fluor 488 (Invitrogen; A-21131) |
| Cultured cells | **3 d** | Rabbit IgG anti **PDGFRα**  (1:500) (SantaCruz; sc-338)    Mouse IgG1 anti-**BrdU** antibody (1:200, Sigma; B8434) | Goat anti-rabbit IgG (H+L) Alexa Fluor 546 (Invitrogen; A-11035)    Goat anti-mouse IgG1 Alexa Fluor 488 (Invitrogen; A-21121 |
|  | **1-3 d** | Rabbit IgG anti-**OLIG2** (1:1000)  (MERCK; ABN899)    Mouse IgG1 anti-**Ki67** (1:100)  (Novocastra; NCL-L-Ki67-MM1) | Goat anti-rabbit IgG Alexa Fluor 488 (Invitrogen; A-11008)    Goat anti-mouse IgG1 Alexa Fluor 546 (Invitrogen; A-21123) |
|  | **3 d** | *Mouse IgG3 anti-**GalC** (1:200) (Chemicon; MAB342) | Goat anti-mouse IgG (H+L) Alexa Fluor 546 (Invitrogen; A-11003) |

*Triton X-100 was not added to the blocking mixture for in vitro cell staining according to the manufacturer's instructions

**Supplementary Table 2.** **List of primer pair sequences** **for the use in qPCR analysis.**

| Tested gene | Primer sequences | |
| --- | --- | --- |
| Ascl1 | F: 5’ GGAACAAGAGCTGCTGGACT 3’ | R: 5’ CTCCCCCTTTTGACGTCGTT 3’ |
| Cnp | F: 5’ GACCTGGTCAGCTATTTTGGC 3’ | R: 5’ GGCCTTGCCGTAAGATCTCC 3’ |
| Mag | F: 5’ TCCTGATTGCCATTGTCTGCT 3’ | R: 5’ AGAGATTCGGAATTCGGGGC 3’ |
| Mbp | F: 5’ GCCTGTCCCTCAGCAGATTT 3’ | R: 5’ GTCGTAGGCCCCCTTGAATC 3’ |
| Myrf | F: 5’ GTGGTACTGTGCAGCCTGAT 3’ | R: 5’ CACGGAAGGACAGGATGGTT 3’ |
| Nkx2.2 | F: 5’ ACCGAGGGCCTCCAATACT 3’ | R: 5’ GTCTCCTTGTCATTGTCCGGT 3’ |
| Olig1 | F: 5’ TCTGTCTTTCAGGCTCGCAC 3’ | R: 5’ AAGTCCAGAACACCGATGGC 3’ |
| Olig2 | F: 5’ AGCGAGCACCTCAAATCGAA 3’ | R: 5’ AAGATCATCGGGTTCTGGCG 3’ |
| Plp | F: 5’ TTGCCTTCCCTAGCAAGACC 3’ | R: 5’ CTCGGCTGTTTTGCAGATGG 3’ |
| Sox10 | F: 5’ GGCTCACTACAAGAGTGCCC 3’ | R: 5’ CTGTCTTTGGGGTGGTTGGA 3’ |
| B2m | F: 5’ CGGGGTGGTGATGAGAAGTT 3’ | R: 5’ AAGGCTCCTTGTCCCTTGAC 3’ |
| Rpl13 | F: 5’ GAAGAAGGGAGACAGTTCTGC 3’ | R: 5’ AGTTCTTCTCCTCTTCCGTGATG 3’ |

Primers were designed with Primer-BLAST software. The primer pairs were characterised by the following properties: primer pairs separated by at least 1 intron in the corresponding genomic DNA (except Olig2 and B2m), length of primers 19-23 bases, PCR product length 75-150 bases, melting temperature of the primers 59-60 ^o^C. The specificity of the designed primer pairs was then verified in the Nucleotide BLAST software.

**
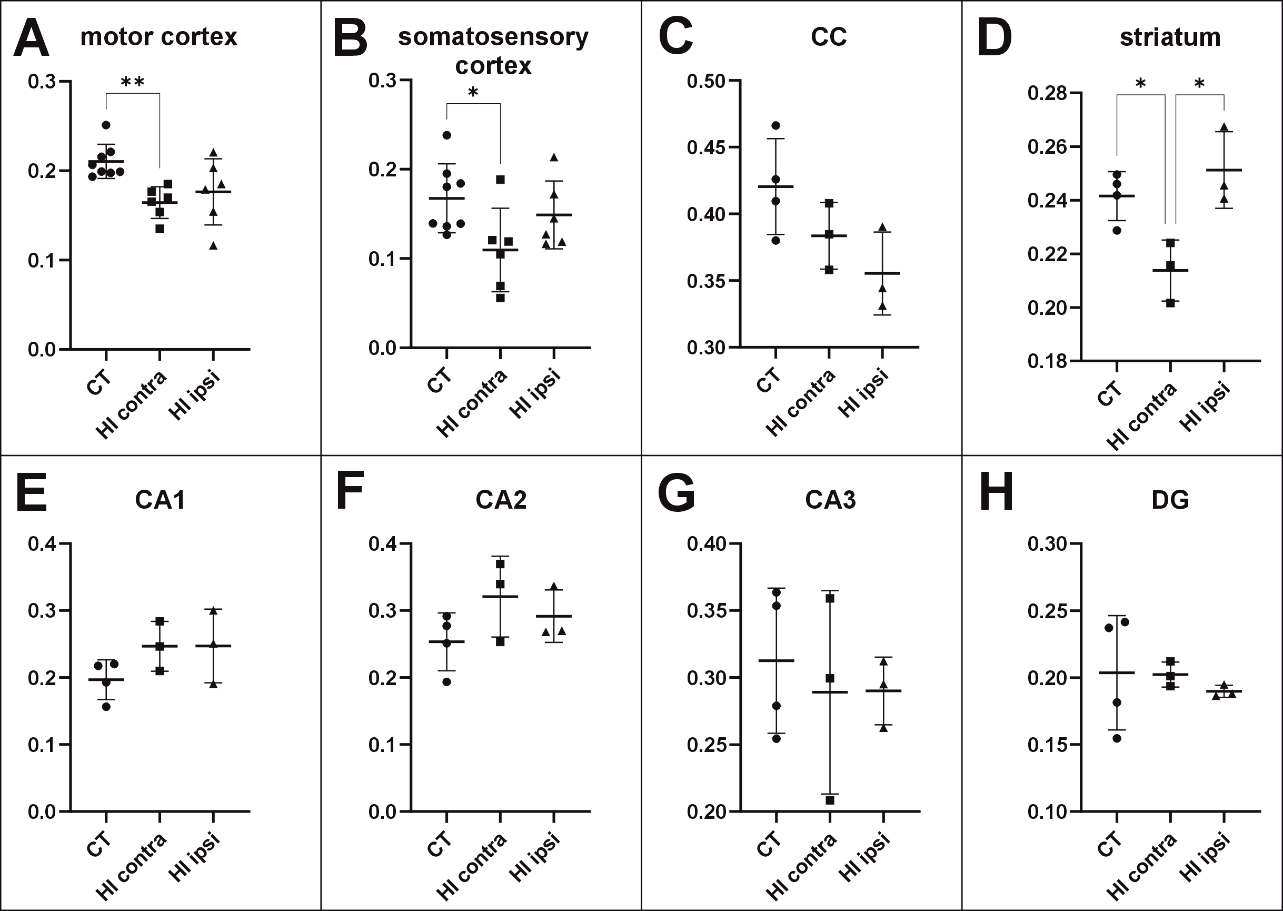
Supplementary Figure 1.**

**Supplementary Figure 1. The results of the FA absolute values in selected brain regions 10 weeks after HI.** Analyzed regions included motor cortex (A), somatosensory cortex (B), corpus callosum (C), striatum (D), CA1 of the hippocampus (F), CA2 of the hippocampus (G), CA3 of thehippocampus (H) and DG of the hippocampus (I). Number of control animals N=2; number of post-HI animals N=3. Dots on the graphs represent single measurement, middle line represent mean value and whiskers represent standard deviation. Normal distribution was evaluated with Shapiro-Wilk test. Statistical significance of the obtained data with normal distribution was assessed by one-way ANOVA with Bonferroni correction (**A**-**E**; **G**-**H**). Statistical significance of the obtained data without normal distribution (**F**) was assessed by Kruskal-Wallis test with Dunn’s correction. Statistically significant differences *p<0.05; **p<0.01.
